# Suppression of *Escherichia coli* Growth Dynamics via RNAs Secreted by Competing Bacteria

**DOI:** 10.3389/fmolb.2021.609979

**Published:** 2021-04-15

**Authors:** Natalia Markelova, Olga Glazunova, Olga Alikina, Valeriy Panyukov, Konstantin Shavkunov, Olga Ozoline

**Affiliations:** ^1^Laboratory of Functional Genomics and Cellular Stress, Institute of Cell Biophysics of the Russian Academy of Sciences, Pushchino Scientific Center for Biological Research of the Russian Academy of Sciences, Pushchino, Russia; ^2^Department of Structural and Functional Genomics, Pushchino Scientific Center for Biological Research of the Russian Academy of Sciences, Pushchino, Russia; ^3^Laboratory of Bioinformatics, Institute of Mathematical Problems of Biology, Pushchino, Russia

**Keywords:** secreted RNAs, RNA-mediated bacterial communications, transcription, *Prevotella copri*, *Rhodospirillum rubrum*, 5′/3′-specific RNA-Seq

## Abstract

With the discovery of secreted RNAs, it has become apparent that the biological role of regulatory oligonucleotides likely goes beyond the borders of individual cells. However, the mechanisms of their action are still comprehended only in general terms and mainly for eukaryotic microRNAs, which can interfere with mRNAs even in distant recipient cells. It has recently become clear that bacterial cells lacking interference systems can also respond to eukaryotic microRNAs that have targets in their genomes. However, the question of whether bacteria can perceive information transmitted by oligonucleotides secreted by other prokaryotes remained open. Here we evaluated the fraction of short RNAs secreted by *Escherichia coli* during individual and mixed growth with *Rhodospirillum rubrum or Prevotella copri*, and found that in the presence of other bacteria *E. coli* tends to excrete oligonucleotides homologous to alien genomes. Based on this observation, we selected four RNAs secreted by either *R. rubrum* or *P. copri*, together with one *E. coli*-specific oligonucleotide. Both fragments of *R. rubrum* 23S-RNA suppressed the growth of *E. coli*. Of the two fragments secreted by *P. copri*, one abolished the stimulatory effect of *E. coli* RNA derived from the 3′-UTR of ProA mRNA, while the other inhibited bacterial growth only in the double-stranded state with complementary RNA. The ability of two RNAs secreted by cohabiting bacteria to enter *E. coli* cells was demonstrated using confocal microscopy. Since selected *E. coli*-specific RNA also affected the growth of this bacterium, we conclude that bacterial RNAs can participate in inter- and intraspecies signaling.

## Introduction

High-throughput sequencing techniques have provided a favorable environment for cellular transcriptome characterization and a much deeper investigation of the RNA world (Forde and O'Toole, 2013; Kukurba and Montgomery, 2015). A great many of transcripts with poorly studied or unobvious functions have been identified in all types of organisms, and the diversity of their roles raises numerous questions in terms of structure and function to be addressed (Chen and Conn, 2017; Leighton and Bredy, 2018; Rosace et al., 2020). For instance, in eukaryotes particular non-coding RNAs (ncRNAs) are known to mediate epigenetic modifications (Castel and Martienssen, 2013), are involved in chromatin remodeling (Nozawa et al., 2017) and participate in the DNA damage response (d'Adda di Fagagna, 2014). Yet, it is not clear how crucial RNA molecules are in these processes. Comprehensively studied systems of higher organisms employing microRNAs (21−25 nt in length) for mRNAs degradation (Miyoshi et al., 2016; Nakanishi, 2016) have not been registered in prokaryotes so far, though bacteria possess single-stranded RNAs sized 15-26 nt (Lee and Hong, 2012; Kang et al., 2013; Bloch et al., 2017). The unique CRISPR-Cas systems of prokaryotes can be considered as functional analogs of eukaryotic small interfering RNAs (siRNAs), mediating protection of the cells against viruses and mobile genetic elements (Obbard et al., 2009; Levanova and Poranen, 2018). Using different mechanisms, both of them rely on RNA sequence specificity.

Many cellular oligonucleotides are products of endonuclease cleavage (Davis and Waldor, 2007; Papenfort et al., 2009; Chao et al., 2017), and some short fragments of tRNAs are proven to be functional (Lalaouna et al., 2015a,b; Diebel et al., 2016; Swiatowy and Jagodzińśki, 2018). In recent years it has been also witnessed for both bacteria and higher organisms that 3′-terminal regions of mRNAs can also be sources of short regulatory RNAs specifically processed by RNAse E (Eisenhardt et al., 2018; Miyakoshi et al., 2019; Hoyos et al., 2020; Wang et al., 2020). Thus, it is likely that the processing of many RNAs with well-established function plays a specific role in living cells by providing short oligonucleotides for diverse regulatory networks.

It is well-known that eukaryotic microRNAs are abundant in the blood, urine and other specimens (Beatty et al., 2014; Fritz et al., 2016; Wiegand et al., 2018; Yau et al., 2019), where they exhibit high stability (Mall et al., 2013; Glinge et al., 2017), in particular because of the protective effect of exosomes (Cheng et al., 2014). Similarly, prokaryotic small RNAs (exRNAs) have been identified in extracellular media (Ghosal et al., 2015; Blenkiron et al., 2016; Alikina et al., 2018), being protected from cleavage due to incorporation into outer membrane vesicles (Kulp et al., 2015; O'Donoghue and Krachler, 2016; Malabirade et al., 2018), or through complex formation with proteins. If excreted from the cells via vesicular transport, bacterial RNAs can be internalized into the cells of a host, leading to either upregulation (Mills, 2011; Abdullah et al., 2012) or suppression (Koeppen et al., 2016; Ahmadi Badi et al., 2020) of its innate immune system. The main mechanism ensuring discrimination of incoming alien tRNAs and rRNAs from domestic molecules resides on the activity of intracellular type 7 and 8 Toll-like receptors (TLRs) of eukaryotic cells, which recognize single-stranded oligonucleotides with typical for bacteria poly-U- and GU-tracks (Heil et al., 2004; Eigenbrod et al., 2015). TLR7 can be activated by different RNAs, while TLR8 senses fragments of 23S rRNA with UGG, UAA and UGA motifs (Krüger et al., 2015), as well as ultra-short oligonucleotides UG and UUG (Geyer et al., 2015). Unlike type 8 receptors, TLR7 are sensitive to ribose methylation in particular positions of tRNAs, which prevents the development of immune reaction to the host tRNAs (Kaiser et al., 2014; Jung et al., 2015). This exemplifies that existing under conditions of constant close interaction with microbes, eukaryotes have developed an elaborate system for fine and specific sensing of bacterial transcripts.

Microbes inhabiting the intestine are able to affect the spectrum of the host intracellular microRNAs, which in turn influence the expression of the host genes (Dalmasso et al., 2011). Vice versa, it has been demonstrated that synthetic copies of particular mouse/human microRNAs from epithelial cells added *in vitro* to the cultures of *Fusobacterium nucleatum* and *Escherichia coli* shape the expression of certain genes through formation of complementary duplexes, promoting bacterial growth (Liu et al., 2016). Moreover, the population of various microRNAs, produced by the gut epithelial cells has been proven to be critical in shaping the composition of the microbiome. Considering the discovery of Ago protein homologs (Willkomm et al., 2015) and the presence of RNase III family enzymes required for processing in bacteria (Bechhofer and Deutscher, 2019), it is reasonable to expect that prokaryotes are also able to “perceive” the RNA-encoded information from other bacteria. However, so far only the circumstantial evidence discussed above supports this possibility, and there are no model exRNAs that could be used for detailed studies. The main complication in conducting such studies is related to the short length of exRNAs, which requires special efforts to distinguish between RNAs secreted by each type of biological objects in mixed populations. Here we applied differential analysis to visualize changes in the RNA secretomes of *E. coli* in response to the presence of *P. copri* and *R. rubrum*. We also used sets of short oligonucleotides (*k*-mers, *k* = 16, 18, 20, or 22), either unique or common, in the genomes of *E. coli* and two model bacteria, to deduce which of them are predominantly secreted by *E. coli* in response to the presence of another bacterium. This allowed us to identify potentially active exRNAs and for the first time demonstrate the ability of their synthetic analogs to penetrate into *E. coli* cells and influence their growth.

## Materials and Methods

### Bacterial Strains and Growth Conditions

RNA secretion was studied for a laboratory strain of *Escherichia coli* K12 MG1655 (*E. coli*). *Rhodospirillum rubrum* ATCC 11170 (*R. rubrum*) and *Prevotella copri* (*P. copri*) obtained from the DSMZ collection (DSM 467 and DSM 18205, respectively, https://www.dsmz.de/catalogs/parts/culture), were used only as representatives of intestinal microflora, capable of provoking an adaptive response of *E. coli*. Since *P. copri* is an obligate anaerobe, bacteria were grown at a low oxygen concentration (1−1.5%) in all experiments. To control the oxygen level before inoculation, resazurin (1 mg/l) was added to all media.

Oxygen was removed from culture media by gentle heating and rapid cooling in a flow of CO_2_. Before cultivation, empty Hungate tubes were treated with a stream of N_2_ gas for several minutes, after which they were filled with required volumes of media, again kept under N_2_ flow for several minutes, immediately sealed and sterilized (121°C, 20 min). The pH of the medium was controlled before and after autoclaving. To prevent exRNAs from degradation and favor conditions of rhodospirilla growth (Kaiser and Oelze, 1980), bacteria were cultured at 30°C. Cells were cultured for 8.5 h after inoculation, when the optical density of all individually grown cultures was approximately the same (Supplementary Figure 2). Bacterial cells were precipitated by centrifugation at 3,000 rpm (4°C), after which the required volume of supernatant was withdrawn with a syringe. In case when complete removal of cells was required, the medium was filtered twice using Millipore filters with a pore diameter of 0.22 μm and then used for RNA isolation.

Conditions for co-culturing were selected based on preliminary data obtained using a Synergy H1 Hybrid Multi-Mode Reader (BioTek Instruments, USA) with plastic 96-well suspension plates (TC Plate Well, Suspension, F; Sarstedt). Bacterial growth was initiated by inoculation according to the Hungate technique (Hungate, 1969; Wolfe, 1971) with stationary phase cultures (overnight growth for *E. coli* and 48-h cultivation for *P. copri* and *R. rubrum*). The cultures were inoculated anaerobically in 10 ml medium. *E. coli* inoculum was serially diluted and added in 250 μl volume so as to obtain the final dilution ratio of stationary phase cultures 1:2000 or 1:4000 (Supplementary Figures 1, 2). The final ratios for *P. copri* and *R. rubrum* were 1:20 and 1:10, respectively, as chosen empirically between 1:10, 1:20, and 1:50 to obtain approximately the same optical density in individual cultures at the time point of harvesting (OD_600_ = 0.4). Inoculum volumes in these cases varied from 1 ml to 200 μl. Following inoculation, 0.2 ml of prepared cultures were pipetted in the plate wells for incubation in the reader. Examples of dynamic curves for individual and mixed cultures grown in parallel at 30°C are shown in Supplementary Figure 2.

Since *R. rubrum* cannot grow on M9 mineral medium [KH_2_PO_4_-3 g/l, Na_2_HPO_4_−6 g/l, NaCl−0.5 g/l, NH_4_Cl−1 g/l, L-cysteine 0.04 g/l, D-glucose−0.5%, 2 mM MgSO_4_, CaCl_2_−0.1 mM (pH = 7.0)], three other media were tested to obtain a comparable growth rate for model bacteria (Supplementary Figure 1). Schaedler Anaerobe Broth (Oxoid CM0497) provided a highly efficient growth of all strains (Supplementary Figure 1A). However, in addition to mineral components [glucose−5 g/l, cysteine HCl−0.4 g/l, hemin−0.01 g/l, 0.75 g/l Tris buffer (pH 7.6 at 25°C)], and commonly used nutrients (peptone−5 g/l, yeast extract−5 g/l), this broth also contains 10 g/l of Tryptone Soya Broth (Oxoid CM129), which adds plant nucleic acids to the set of contaminating molecules.

Supplement of 0.01 g/l hemin required for prevotella and 0.3% glucose to a very complex medium optimized for rhodospirilla (yeast extract−0.3 g/l; Na_2_-succinate−1 g/l; NH_4_-acetate−0.5 g/l; 0.1% solution of Fe(III) citrate−5 ml/l; KH_2_PO_4_-0.5 g/l; MgSO_4_ x 7 H_2_O−0.4 g/l; NaCl−0.4 g/l; NH_4_Cl−0.4 g/l; CaCl_2_ x 2 H_2_O−0.05 g/l; 0.01% solution of vitamin B12–0.4 ml/l; L-cysteine chloride−0.3 g/l; 0.1% resazurin−0.5 ml/l; Trace element solution SL-6–1 ml/l. Composition of SL-6: ZnSO_4_ x 7 H_2_O−0.1 g/l; MnCl_2_ x 4 H_2_O−0.03g/l; H_3_BO_3_-0.3 g/l; CoCl_2_ x 6 H_2_O−0.2 g/l; CuCl_2_ x 2 H_2_O−0.01g/l; NiCl_2_ x 6 H_2_O−0.02 g/l; Na_2_MoO_4_ x 2 H_2_O−0.03 g/l, pH = 6.8) allowed to obtain a comparable growth rate for all the three strains (Supplementary Figure 1B). However, the dynamics of growth was very slow, which increased the risk of accumulation of RNA degradation products. Therefore, we used modified Luria-Bertani broth (LB), containing peptone (10 g/l); yeast extract (0.5 g/l); NaCl (10 g/l); L-cysteine HCl (1 g/l); and resazurin (1 mg/l), as a reasonable compromise between a high growth yield and a low level of impurities in the sample (Supplementary Figure 1C).

### RNA Purification and Sequencing

Bacteria for RNA purification were grown in tightly closed Hungate tubes in 10 ml LB medium (pH=7.0) or LB medium pretreated with 5 M NaOH (Table 1). To control anaerobic state, all cultures were grown with 1 mg/l resazurin. To account for extraneous transcripts, fraction of short RNAs was extracted from LB medium using Qiagen miRNeasy Serum/Plasma kit (Qiagen, Germany) (sample LB_1_medium in Table 1). For sample LB_2_medium (Table 1), fraction of short RNAs was extracted from LB medium reversibly treated with 5M NaOH, as proposed by Pavankumar et al. (2012) using Qiagen miRNeasy Serum/Plasma kit (Qiagen, Germany). Intracellular RNAs were isolated from 0.5 ml cultures as described previously (Bykov et al., 2016; Alikina et al., 2018) with TRIzol RNA extraction reagent (Invitrogen, USA) and the sample was enriched for short RNAs using mirVana miRNA Isolation Kit (Ambion, USA). The concentration of RNA samples was estimated using Nanopore ND-1000 and Qubit 3 (Thermo Fisher Scientific, USA). For extraction of extracellular (secreted) RNAs, 1 ml of cultures were centrifuged for 15 min at 3,000 RCF, followed by supernatant filtration using a sterile syringe and two simultaneously mounted 0.22 mkm filters, followed by purification of short RNA fraction using Qiagen miRNeasy Serum/Plasma kit (Qiagen, Germany). A set of RNAs composed of those detected in either treated or untreated media was used to remove environment-attributed sequences from the resulting ensembles of sequence reads.

**Table 1:** Sequencing statistics of RNA samples.

Sample^*^	**Number of reads**		**Notes**
	**Before QC**	**After QC**	
First set (LB-medium without NaOH treatment)			
LB_1_medium	99,347	75,354	
Eco_out_1	1,677,610	1,484,046	RNAs secreted by *E. coli* in monoculture
Eco_Prevot_1	1,532,963	1,290,466	RNAs secreted by *E. coli* and *P. copri* in mixed population
Eco_Rhod_1	1,625,146	1,429,628	RNAs secreted by *E. coli* and *R. rubrum* in mixed population
Second set (LB-medium without NaOH treatment)			
Eco_out_2	1,168,144	846,669	RNAs secreted by *E. coli* in monoculture
Eco_Prevot_2	937,975	662,605	RNAs secreted by *E. coli* and *P. copri* in mixed population
Eco_Rhod_2	807,732	644,415	RNAs secreted by *E. coli* and *R. rubrum* in mixed population
Third set (NaOH treated LB)			
LB_2_medium	2,728,221	2,034,497	
Eco_in	1,320,485	299,167	Intracellular RNAs isolated from *E.coli* monoculture
Eco_Prevot_3	2,377,939	2,097,966	RNAs secreted by *E. coli* and *P. copri* in mixed population
Eco_Prevot_4	711,408	447,987	RNAs secreted by *E. coli* and *P. copri* in mixed population
Eco_Rhod_3	2,123,797	998,024	RNAs secreted by *E. coli* and *R. rubrum* in mixed population

* *All sequence reads libraries are available at https://www.ncbi.nlm.nih.gov/bioproject/PRJNA687658)*.

Libraries for sequencing were prepared using Ion Total RNA-Seq Kit v2 (Thermo Fisher Scientific, USA) according to the protocol of the manufacturer with modifications. MicroRNA samples were subjected to adapter ligation and reverse transcription according to the protocol, then cleaned using Monarch PCR & DNA Cleanup Kit (New England Biolabs, USA) and fractionated in 6% polyacrylamide gel with subsequent staining with ethidium bromide. Pieces of gel containing DNA fragments of required length (90-123 bp) were cut out, minced and eluted overnight in Elution Buffer from NEBNext Multiplex Small RNA Library Prep Set for Illumina (Set 1) (New England Biolabs). Then ammonium acetate (final concentration of 0.3 M) and linear acrylamide solutions were added to DNA-containing supernatant, followed by 2-h precipitation with absolute ethanol at −80°C and centrifugation at 15,000 RCF (−4°C). The pellet was washed with 80% ethanol, dried at RT for 5–10 min, dissolved in TE buffer from NEBNext Multiplex Small RNA Library Prep Set for Illumina (Set 1) and amplified according to the protocol for Ion Total RNA-Seq Kit v2. The sample was then purified using Nucleic Acid Binding Beads from Ion Total RNA-Seq Kit v2, dissolved in pre-warmed (37°C) nuclease-free water, assessed for concentration using Qubit 3, diluted to 100 pM and used for emulsion PCR on Ion OneTouch^TM^ 2 System with Ion PGM Hi-Q View OT2 Kit (Thermo Fisher Scientific). Sequence reads libraries obtained with Ion Torrent PGM analyzer (Thermo Fisher Scientific) are available in https://www.ncbi.nlm.nih.gov/bioproject/PRJNA687658.

### Bioinformatic Analysis

The genomes of *E. coli* K12 MG1655 (Blattner et al., 1997) and *R. rubrum* ATCC 11170 (Munk et al., 2011) were taken from the NCBI RefSeq database (accession numbers NC_000913.3 and NC_007641, respectively). The genome of *Prevotella copri* DSM 18205 was obtained from NCBI GenBank (project NZ_ACBX00000000.2, direct submission). Sequence reads were quality filtered (QC) with Filter by Quality tool on Galaxy server (Afgan et al., 2016) using Q15 as the threshold level for nucleotides (*p* < 0.036) and requiring at least 90% presence of such nucleotides in reads. The obtained nucleotide sequences were sorted by size [12–50 nucleotides (n)] and fragments with sequences found in the LB medium were removed from the data sets. Filtered libraries were mapped onto the *E. coli* genome using Matcher algorithm (http://www.mathcell.ru/DnaRnaTools/Matcher.zip) as described previously (Panyukov et al., 2013; Antipov et al., 2017; Alikina et al., 2018). In brief: fragments of each size were evaluated separately, requiring precise matching to the genome. The sequences were assigned to the positions corresponding to the 5′-ends of reads mapped to the top strand of the genome and to the positions corresponding to the 3′-ends if reads were mapped to the bottom strand. Reads with multiple entries in the genome were assigned to all such sites in equal proportion.

The proportion of species-specific RNAs and oligonucleotides common to co-habiting bacteria in the fraction of secreted RNAs was estimated using *k*-mers present in the genome of *E. coli* and either absent or present in the genomes of the competing bacteria. Sets of *E. coli*-specific *k*-mers (*k* = 16, 18, 20, 22) were obtained with UniSeq software (Panyukov et al., 2017, 2020). Sets of common *k*-mers of the same length were collected using a simpler program that sorted all *k*-mers in lexicographic order and searched for those that have copies in the genome of comparison.

### Functional Analysis of Selected Oligonucleotides

The candidate oligonucleotides were synthesized and purified by chromatography at Syntol (Russia). They were dissolved in sterile nuclease-free water to a concentration of 100 nmol (stock solution). Their effect was assessed in plastic 96-well suspension plates (TC Plate Well, Suspension, F; Sarstedt) using Synergy H1 Hybrid Multi-Mode Reader (BioTek Instruments, USA). Oligonucleotides were added to bacterial cultures inoculated at a ratio of 1:500 immediately before cultivation in M9 medium. This minimal medium, which supports sufficient growth rate of *E. coli*, was chosen for these functional tests so that to avoid possible interaction between the oligoribonucleotides studied and the inevitable transcripts from the components of rich media, such as yeast extract or peptone. In a pilot experiment carried out with *rrs*_−115_, _C_tRNA and ProA-ter, 1 and 2 nmol concentration of oligonucleotides was used. Having found a small dose dependence, we carried out all subsequent experiments with a concentration of 2 nmol, but all samples were used to assess the mean of the observed effects and SEM, because the dose dependence was smaller than the variation between different experiments. Growth dynamics were analyzed using GraphPad Prizm 5 software (Appling, 2008). The area under the growth curves was calculated and the ratio of the values obtained for the experimental and control samples was used as a characteristic for comparison.

### Complementary Duplexes Annealing

Three complementary duplexes were prepared to compare the effects from single-stranded oligonucleotides and their double-stranded forms. They included duplexes of tRNA with _C_tRNA, *rrs*_−115_ with _C_*rrs*_−115_ and ProA-ter with *rrl_Pr*. Oligonucleotides from stock solutions were mixed in equimolar concentrations (20 μl), melted and annealed in DTlite 4 Real-Time PCR System (DNA Technology, Russia) according to the following program: heating from RT at a rate of 1°C/0.8 min; melting for 5 min at 75.2°C in the case of tRNA/_C_tRNA duplex and for 5 min at 56°C in the case of *rrs*_−115_/_*C*_*rrs*_−115_ and ProA-ter/*rrl_Pr* duplexes; cooling to storage temperature (4°C) at a rate of 1°C/10 min. Single stranded oligonucleotides and their duplex were electrophoretically analyzed in 15% PAGE at 150V using 50+ bp DNA Ladder (Evrogen, Russia) as markers. Freshly prepared duplexes were added to the bacterial cultures to a final duplex concentration of 2 nmol.

### Confocal Microscopy

*E. coli* K12 MG1655 cells were tested for the ability to uptake extraneous RNA molecules. Selected RNA molecules (*rrl*_2585 and *rrl*_Pr) were synthesized and labeled with Cy5 on the 3′-ends (Syntol, Russia). Cells were cultured in 0.5 ml of LB medium at 30°C, 32°C or 37°C for 4 h, 8.5 h or 17 h with shaking in the presence of individual Cy5-labeled oligoribonucleotides added to a final concentration of 10 μM at the moment of inoculation. Thirty minutes before the end of culturing, MitoTracker™ Green FM (Thermo Fisher Scientific, USA) was added (2 μM) to provide for cell membrane staining. Following incubation, cells were harvested by centrifugation at room temperature (2,500 RPM) and twice washed with sterile phosphate buffered saline (PBS) to reduce the background fluorescence. After that, pelleted cells were resuspended in 20–50 μl of melted 0.8% agarose; 10 μl of suspension was placed on glass slides and pressed by coverslips. Fluorescent confocal microscopy imaging was obtained on a Leica DMI 6000 CS microscope (Leica, Germany) with a TCS SP5 scanner (Leica, Germany) and LAS X Software (Leica, Germany) with excitation/emission at 640/690 nm and 500/550 nm for Cy5 and MitoTracker™ Green FM, respectively.

### Statistics

SigmaStat one-sample *t*-test of SigmaPlot 6 software package was used to calculate SEM values for variables of single groups and the option “compare two groups” was applied to estimate *p*-values of differences (http://www.sigmaplot.co.uk/products/sigmaplot/statistics.php). The percentages of *E. coli*–specific *k*-mers and oligonucleotides with similar sequences in the genomes of *E. coli* and two competing bacteria were estimated for 16−, 18−, 20−, and 22−mers in 2−4 sequencing libraries listed in Table 2 with subsequent averaging of 8−16 values. The functionality of the selected oligonucleotides was evaluated based on 3−9 growth experiments with three technical replicates in each. Deviations from control cultures that grew in M9 medium without model oligonucleotides were averaged for similar samples in the experiment and the average value between all experiments was used for comparison. The number of biological repeats is indicated in **Figure 3**.

**Table 2:** Numbers of unique and common *k*-mers in the genome of *E. coli* assessed pairwise with the genomes of *P. copri* and *R. rubrum*.

**Type of sequences**	***k* = 16**	***k* = 18**	***k* = 20**	***k* = 22**
Number of *E. coli* unique *k*-mers				
Absent in *P. copri*	8,960,945	9,023,619	9,032,272	9,035,542
Absent in *R. rubrum*	8,931,481	9,018,843	9,030,542	9,034,516
Number of *k*-mers present in the genomes of *E. coli* and competing bacteria				
Overlap with *P. copri*	28,748	2,854	676	376
Overlap with *R. rubrum*	53,512	6,942	2,298	1,360
Number of *k*-mers present in all the three genomes				
Present in *E. coli, P. copri, R. rubrum*	778	366	286	222

## Results

### *P. copri* and *R. rubrum* Affect the Profile of RNAs Secreted by *E. coli*

The main goal of the study was to find oligonucleotides that can affect population homeostasis of *E. coli*. Therefore, we used two bacteria from the human intestinal microbiome, *P. copri* and *R. rubrum* as natural partners of *E. coli* with presumably established RNA-mediated communication systems. According to the ENCODE project, *E. coli* is in antagonistic relationships with both bacteria, which, on the contrary, exhibit mutual symbiosis (Arumugam et al., 2011). The data of our growth experiments are consistent with both these conclusions, since the optical density in the mixed populations of *E. coli* + *P. copri* and *E. coli* + *R. rubrum* increased slower (Supplementary Figures 2A,B), while in the pair *P. copri* + *R. rubrum* faster (Supplementary Figure 2C) than expected, in the absence of mutual influence of bacterial cultures.

**Figure 1:**
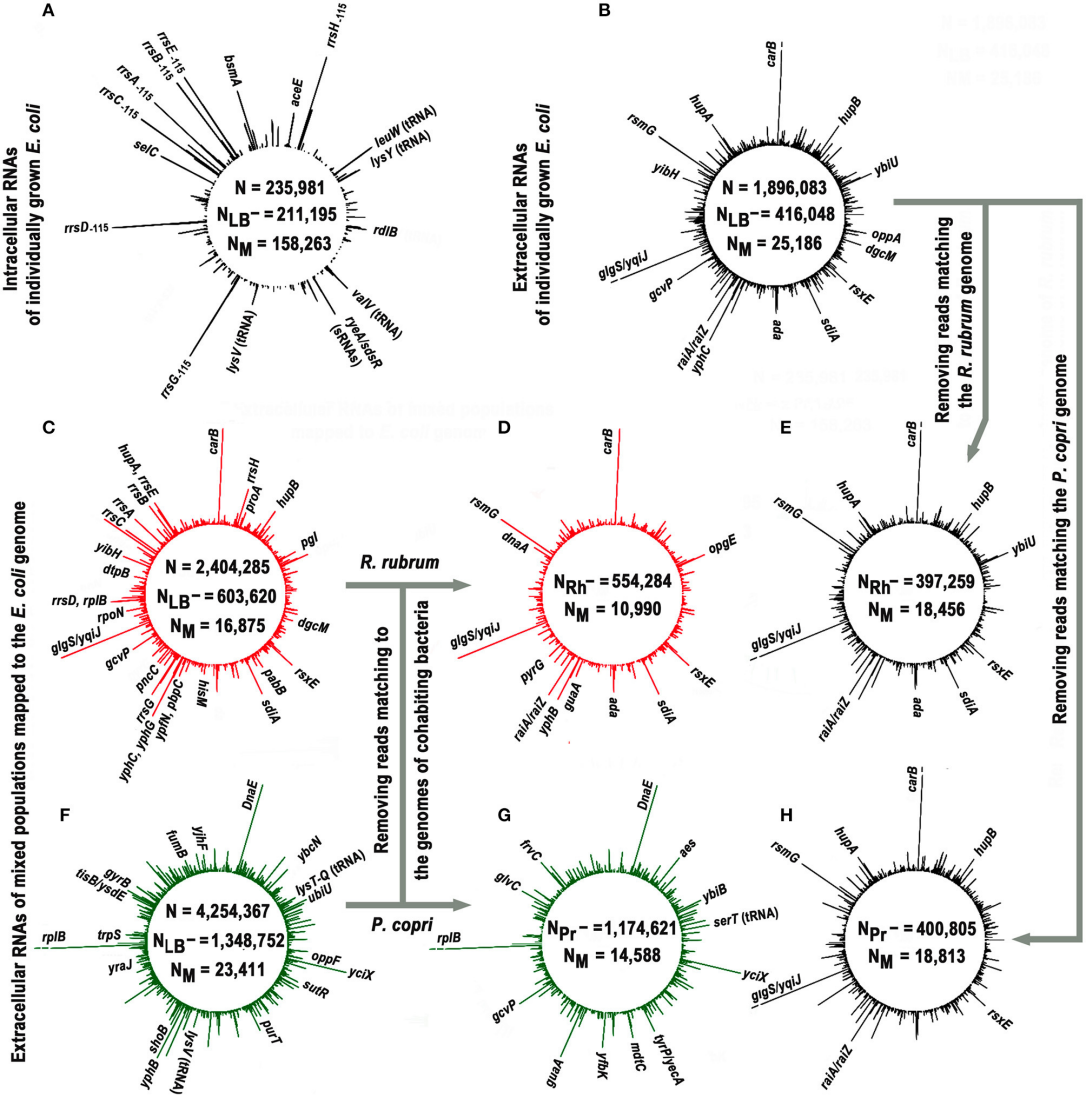
Profiles of short intracellular and secreted (12−50 n) RNAs obtained in monoculture of *E. coli* (black plots) and in its mixed populations with *R. rubrum* (red plots) or *P. copri* (green plots) and mapped to the genome of *E. coli*. The profiles **(A,B)** show the genomic distribution of intracellular (experiment Eco_in) and external RNAs obtained in *E. coli* monocultures (pooled experiments Eco_out_1 and Eco_out_2), respectively. Values N, N_LB−_, and N_M_ in all panels indicate the total number of 12–50 nucleotide reads that passed quality control; number of reads passed the filtering against LB-derived sequences and number of sequences mapped to the *E. coli* genome, respectively. The profiles **(C,F)** show distribution of exRNAs obtained from mixed populations (pooled libraries from three experiments Eco_Rhod_1–3 and four experiments Eco_Prevot_1–4). Profiles **(D,G)** show putative contribution of *E. coli* to the population of RNA molecules detected in mixed populations. They were obtained from the same sequence libraries as profiles **(C,F)** after all reads mapped to the genomes of *R. rubrum* and *P. copri* were removed, and highly represented RNA products that remained unchanged are indicated in these panels. The profiles **(E,H)** were plotted in the same way using the set of oligonucleotides profiled in **(B)**. The values N_Rh−_ and N_Pr_- indicate the number of reads in combined libraries that do not match to the genomes of *R. rubrum* and *P. copri*, respectively. All profiles were drawn with DNAPlotter v. 1.3 (Carver et al., 2009) using a running window of 10 bp and a step size of 1 bp. The scale of such plots is automatically determined by the amplitude of the maximum peak. In profiles with exoRNAs they were equal to 239 **(B,E,H)**, 219 **(C,D)**, and 264 **(F,G)** reads. Therefore, to obtain an equal resolution for other peaks in the profiles, the breaks were made at the level of 219 reads [indicated in **(B,E,H)** for similar products from the coding sequence of the *carB* gene and an intergenic space *glgS/yqiJ*, as well as for fragments of *rplB* aRNA in **(F,G)**].

Nine experiments were carried out to collect RNAs secreted by *E. coli* in conditions of individual and mixed growth with each of the two model bacteria (Table 1). Only reads 12-50 nucleotides (n) long, precisely matching to the genome of *E. coli*, were taken into account (Figure 1). To avoid the contribution of oligonucleotides contaminating the LB environment, independently prepared samples LB_1_medium and LB_2_medium (Table 1) were sequenced. Reads from either of these two experiments were subtracted from all experimental libraries, including the set of intracellular RNAs. This procedure reduced the set of internal RNAs by only 10.1%, while the same filtration made for reads from extracellular samples excluded 78.1% of sequences from further analysis (statistical parameters indicated in Figures 1A,B). This difference in the degree of extraneous oligonucleotides in the extracellular and intracellular samples means that not every RNA molecule appeared in the medium from peptone or yeast extract can penetrate into bacterial cells.

Approximately 75% of intracellular RNAs were precisely mapped to the genome of *E. coli*, while in the case of external RNAs, this fraction was only 6.2% (statistical parameters in Figures 1A,B, respectively). Similar, albeit smaller in scale, losses were observed in extracellular samples obtained from cultures grown on M9 medium (Alikina et al., 2018). Therefore, the residual presence of environmental RNAs, which occasionally escaped sequencing in our control samples, cannot fully explain the observed mapping deficit. Some of the losses are due to various modifications already found in exRNA, including polyadenylation or the addition of other non-template nucleotides to the 3′-ends (Alikina et al., 2018), but most of them are likely due to accidental ligation, since many of non-mapping oligonucleotides are chimeric. In any case, only 25,186 reads from the secreted fraction precisely matching to the *E. coli* genome were collected in two experiments with individually grown *E. coli* (Eco_out_1 and Eco_out_2 samples in Table 1) and were pooled for profiling (Figure 1B).

**Figure 2:**
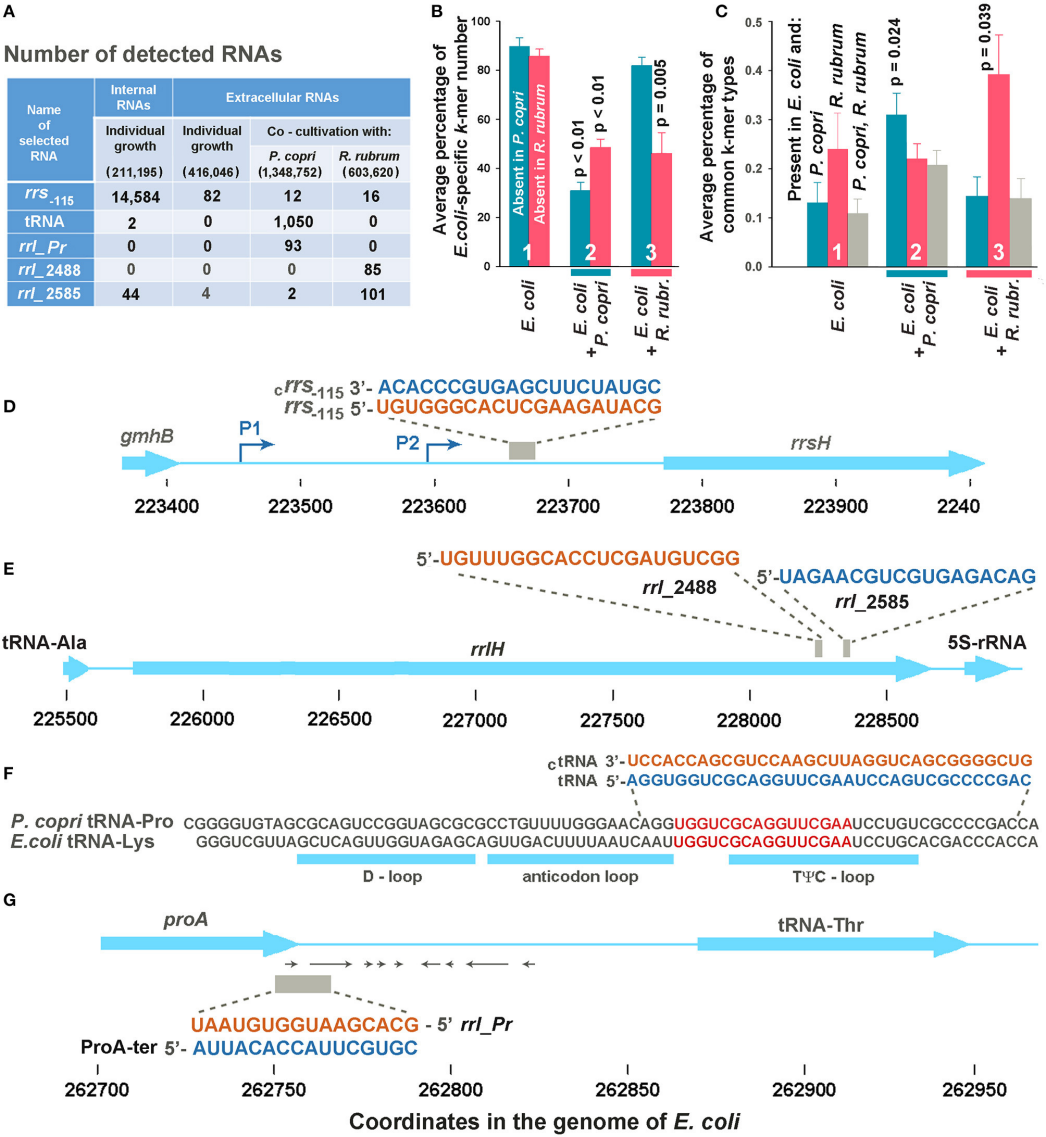
Selection of potentially functional extracellular RNAs. **(A)** Total number of selected *k*-mers found in different RNA fractions in one (Eco_in sample in Supplementary Table 1), two (Eco_out samples 1 and 2), three (Eco_Rhod samples 1-3), and four (Eco_Prevot samples 1-4) experiments. Only reads with a length equal to or longer than the selected *k*-mers were taken into account, but reads with flanking sequences belonging to jointly grown bacteria were not ignored. The total number of reads after quality filtration is indicated in parenthesis. **(B)** Average percentage of *E. coli*-specific *k*-mers in reads mapped to the *E. coli* genome and absent in the genomes of *P. copri* (cyan bars) or *R. rubrum* (magenta bars). **(C)** Diversity of common *k*-mers found in different RNA fractions estimated as the percentage from the total number of *k*-mer types found in the secretomes. Statistical parameters in plots **(B,C)** were calculated for all the sets of *k*-mers obtained in mixed populations, compared with the sets of extracellular *k*-mers secreted by individually grown *E. coli*; however, *p*-values are indicated only for statistically significant changes. **(D,E)** Sequences of samples *rrs*_−115_, *rrl*_2488 and *rrl*_2585 are identical in all seven ribosomal operons of *E. coli*. **(D,E)** Show them in the environment of the H operon. **(F)** Aligned sequences of *E. coli* tRNA-Lys (genes *lysT, lysW, lysY, lysZ, lysQ, lysV*) and *P. copri* tRNA-Pro with the indicated locations of tRNA loops. The selection of two 34-mer oligonucleotides, with flanking sequences from the *P. copri* genome, for experimental evaluation, was based on the presence of 16-mers printed in red. **(G)** Sequence and genomic location of ProA-ter sample transcribed in *E. coli* and its complementary fragment *rrl_pr* from 23S rRNA of *P. copri*. All oligonucleotide sequences are printed in colors corresponding to the color code used in Figure 3.

The profile turned out to be clearly different from the one shown in Figure 1A, which exemplifies the distribution of intracellular oligonucleotides over the genome (Eco_in sample in Table 1). Being consistent with our previously published data for *E. coli* MG1655 grown aerobically in M9 medium (Alikina et al., 2018) and the data obtained by other authors (Ghosal et al., 2015, Blenkiron et al., 2016), this difference is considered as an evidence of the nonrandom selection of RNAs for secretion. The largest contribution to the set of dominant intracellular oligonucleotides (Figure 1A; Supplementary Table 1) is given by RNA fragments originated from genes of tRNAs, 5S RNAs, and large ribosomal RNAs encoded by seven operons (65.8%), while 48.1% of dominant exRNAs were derived from mRNAs and 36.5% from antisense RNAs (Figure 1B; Supplementary Table 2). For instance, oligonucleotides from genes *carB, ybiU, oppA, sdiA, ada, yphC*, and *gcvP* are fragments of mRNAs; products from *yibH* corresponds to its 3′-UTR, while products marked as *hupB, dgcM, rsxE*, and *hupA* match the antisense strand of protein coding genes. As in the previous study (Alikina et al., 2018), most of the registered mRNA fragments can be products of RNase processing. Others can be transcribed as independent transcriptional units from intragenic/intergenic promoters predicted *in silico*, including multiple transcriptional start points (TSP) of promoter islands (PI) (indicated in Supplementary Table 2). Multiplicity of oligonucleotides corresponding to the antisense transcripts (aRNAs) among exRNAs is also in line with the previous data (Alikina et al., 2018). Approximately 42% of them have predicted promoters for transcription. Since in the intracellular fraction we found less than half of the aRNAs that are dominant outside, and only in single or several copies, it is likely that some of them are specifically produced for secretion. Based on this analysis, we chose an oligonucleotide from the leader sequence of 16S rRNAs (*rrs*_−115_ in Figure 2A) for experimental assessment. RNA products from this region of ribosomal operons formed seven peaks with a maximal amplitude in the profile of intracellular short RNAs (Figure 1A; Supplementary Table 1), but had more than 175-fold lower relative abundance in the fractions of exRNAs (Figure 2A). This dramatic decrease made the involvement of *rrs*_−115_ in cell-to-cell communications unlikely, but not excluded, and we checked this in growth experiments.

The search for RNAs involved in interspecies communication assumes the detection of specific changes in the spectrum of oligonucleotides secreted in the presence and absence of competing bacteria. Figures 1C,F show the profiles of exRNAs obtained from mixed populations with *R. rubrum* or *P. copri* and mapped to the genome of *E. coli*. Both obviously differ from the profile of *E. coli* extracellular RNAs shown in Figure 1B. For instance, many fragments of 16S rRNA, different from *rrs*_−115_, which were detected only at the background level inside and outside of individually grown *E. coli* cells, became abundant in the presence of *R. rubrum* (Figure 1C; Supplementary Table 3). Likewise, the fragments of *yfbK* mRNA, *yciX* aRNA and transcripts from *tyrP*/*yecA* intergenic region, undetected at all in experiments with a pure culture of *E. coli*, were detected in a large amount in the presence of *P. copri* (Figures 1F,G, as well as Supplementary Table 4). Since secreted RNAs are short, with a preferable length in the range of about 16-25 nucleotides (Ghosal et al., 2015, Blenkiron et al., 2016; Alikina et al., 2018), many of them map to both genomes in mixed populations. Thus, some of new peaks that appeared in the dominant group in the presence of *R. rubrum* or *P. copri*, may be originated from the transcriptomes of cohabiting bacteria. In order to visualize the individual response of *E. coli* to the presence of competing bacteria, all reads mapped to the genomes of *R. rubrum* or *P. copri* were removed from the libraries obtained from the jointly grown cultures before alignment to the genome of *E. coli* (Figures 1D,G). For direct comparison, a similar procedure was performed for reads obtained from an individually grown population of *E. coli* (Figures 1E,H). The fourth column in Supplementary Table 2, containing a list of 104 genomic regions producing most abundant exRNAs, shows corresponding changes in their number, which in 46 regions remained unchanged, while the contribution given by 15 regions was affected by the removal of reads matching to both *R. rubrum* or *P. copri*. All of them belong to coding sequences that are homologous in different bacterial species.

The dominant peaks obtained in the pooled experiments Eco_Rhod_1, Eco_Rhod_2 and Eco_Rhod_3 (Figure 1C; Supplementary Table 3) coincide with 69 peaks of the RNA profile, secreted by *E. coli* only (Figure 1B; Supplementary Table 2). Figure 1D shows the profile obtained after all reads mapped to the *R. rubrum* genome had been removed from these sequence libraries. As a result, we did not observe a significant difference between the profiles of *E. coli*-specific RNAs secreted in the presence (Figure 1D) and absence (Figure 1E) of *R. rubrum*. The dependence on the presence of *P. copri* was more pronounced (Figures 1G,H). In this case, the dominant peaks obtained in the four pooled experiments Eco_Prev_1–4 (Figure 1F; Supplementary Table 4), coincide with only 35 peaks of the RNA profile in Figure 1B. However, the products of all but one of the remaining 69 genomic loci, at least at a low level, were found in the secretome of a pure *E. coli* population. Their large number, therefore, may be caused by induced synthesis in *E. coli* cells or enhanced secretion from them. For instance, the percentage of *dnaE* mRNA fragments, which were not dominant in pure culture and did not match to the genome of *P. copri*, increased by 17.5 times. The contribution of RNAs that are presumably synthesized from the promoter region of *yqiJ*, as well as the percentage of oligonucleotides produced from *atpI/rsmG* intergenic space, on the contrary, decreased by 27.0 and 2.3 times, respectively. Since oligonucleotides from both these regions do not match the *P. copri* genome, this decrease is hardly explained by RNA interference. Among 26 oligonucleotides with an abundance of at least 0.2% in Eco_out samples, which can only be produced by *E. coli*, twenty were also abundant in the mixed population with *R. rubrum* but only eight retained a high level during co-cultivation with *P. copri*. Therefore, it is likely that *E. coli* established more RNA-mediated links with bacteria belonging to the dominant genera in the human gut, than with taxa less represented in this ecological niche.

### In Mixed Populations Bacteria Tend to Secrete Oligonucleotides With Sequences Present in Both Genomes

The data in Figure 1 show that the percentage of exRNAs mapped to the *E. coli* genome (N_M_) in samples obtained from monocultures decreased from 6.1% (Figure 1B) to 4.6-4.7% (Figures 1D,F) after exclusion of reads corresponding to the genomes of *R. rubrum* or *P. copri*. However, in the sets obtained from mixed populations, the percentages of RNAs mapped to the genome of *E. coli* in the libraries filtered from *R. rubrum* (N_Rh−_) and *P. copri* (N_Pr−_) reads were only 1.98% (Figure 1C) and 1.24% (Figure 1E). In both cases this is lower than the expected two-fold decrease in mapping due to the presence of two bacteria in the sample. This indirectly indicated an increase in the percentage of identical sequences in the secretomes of co-cultured bacteria. Therefore, we collected all sequences with length *k* (*k* = 16, 18, 20 and 22 n), which at least once can be found in the genomes of cohabiting bacteria (*E. coli* and *P. copri* or *E. coli* and *R. rubrum*) and also used UniSeq algorithm to identify *k*-mers unique for *E. coli* in each pair. Their numbers with deleted copies from all 16 sets are indicated in Table 2.

It turned out that *E. coli* grown as a monoculture secretes mostly RNAs with sequences absent in the genomes of two other bacteria (Figure 2B, group 1). In the presence of *R. rubrum* the percentage of RNAs mapped to the genome of *E. coli* and absent in the genome of *P. copri* (cross-talk test) was nearly the same as in the monoculture (cyan bar in group 3). However, in the presence of *P. copri* the percentage of such RNAs was significantly lower (cyan bar in group 2). This is what to be expected, if in mixed populations bacteria tend to secrete *k*-mers with sequences present in both genomes. By contributing to the set of identical *k*-mers secreted by *E. coli* in monoculture, *P. copri* automatically reduces the percentage of *E. coli*-specific RNAs in the common secretome. Thus, it was not surprising that in the presence of *R. rubrum* the percentage of RNAs absent in its genome was also lower than in the monoculture of *E. coli* (magenta bar in group 3), although it was not essentially higher in the common secretome of *E. coli* + *P. copri* (magenta bar in group 2).

To characterize the changes in the presence of common *k*-mers in the fractions of exRNAs, we assessed their diversity, rather than multiplicity (Figure 2C) using eight sets of *k*-mers common for the genome pairs *E. coli*-*P. copri* and *E. coli*-*R. rubrum*. Their number is much lower than that of *E. coli*-specific *k*-mers (Table 2). Copies were removed from all the sets of 16, 18, 20, and 22 n long sequence reads found in the secreted fractions in each experiment and the percentage of common *k*-mers with the corresponding lengths in the resulting sets was estimated (Figure 2C). As a direct consequence of the greater number of identical *k*-mers in the *E. coli*-*R. rubrum* pair, as compared to the *E. coli*-*P. copri* pair (Table 2), the percentage of *k*-mers similar with *R. rubrum* was also higher in the *E. coli* monoculture (magenta bar in group 1, Figure 2C). It was the same in the mixed population of *E. coli* with *P. copri* (cross-talk test, group 2) but significantly (*p* = 0.039) greater in the mixed culture with *R. rubrum* (magenta bars in group 3). The same relative changes were observed for the identical *k*-mers in the genomes of *E. coli* and *P. copri*: their proportion remained at the monoculture level in the presence of *R. rubrum* (cross-talk test), but was more than twice increased in the presence of *P. copri* (cyan bars in Figure 2C). *K*-mers representation in all the three genomes (gray bars) was high and roughly corresponded to the percentage of identical *k*-mers with the lowest abundance. Their high number in the mixed population with *P. copri* explains the low percentage of *E. coli*-specific *k*-mers that are absent in *R. rubrum* (magenta bar in group 2 of Figure 2B).

By definition, this type of analysis is completely independent from the copy number of particular *k*-mers. Due to very low percentage of *k*-mers mapped to bacterial genomes from the total fraction of secreted RNAs (Figures 1B–F), it is also virtually independent from the contribution of *E. coli*-specific *k*-mers. Therefore, the data obtained indicate that in mixed populations bacteria tend to secrete oligonucleotides with sequences present in the genome of co-grown bacterium. Based on this observation, we selected additional samples for experimental assessment among such exRNAs.

### Selection of Oligonucleotides for Experimental Verification

Only common 16, 18, 20 and 22 n long *k*-mers found in all experiments with mixed populations of a certain type and absent in any reads of the medium samples were taken for analysis. As a result, we had 11 oligonucleotides found in the *E. coli*-*R. rubrum* medium and 17 potential candidates revealed in the mixed *E. coli*-*P. copri* populations. Two exRNAs from the first set turned out to be 16S rRNA fragments, while all the others were derived from 23S rRNAs. Two of them, located 2,488 and 2,585 bp from the 5′-end of 23S rRNA molecules (samples *rrl*2488 and *rrl*2585 in Figures 2A,E) were selected for experimental testing. Only *rrl*2585 was detected in the secretome and intracellular transcriptome of *E. coli* and exhibited an increase in number in the presence of *R. rubrum* (Figure 2A). Located at a distance of 97 bp from each other in both genomes, the selected exRNAs belonged to the regions differing in seven base pairs. In mixed populations, all fragments containing those discriminatory nucleotides belonged to *R. rubrum* and are not among the dominant peaks listed in Supplementary Table 3.

Thirteen oligonucleotides from the second set were also processed from structured regions of ribosomal RNAs. Seven of them belong to 23S rRNA and six to 16S rRNA. Two oligonucleotides from 23S rRNA were found in both the common secretomes with *P. copri* and *R. rubrum*. According to discriminatory base pairs, both were predominantly produced by *P. copri*. Three of the other four exRNAs found almost exclusively in the mixed culture with *P. copri* were associated with tRNA genes. One of them corresponding to the T*Ψ*C-loop of the *E. coli* tRNA-Lys was selected for further experiments (Figures 1F, 2A,F; Supplementary Table 4). According to Microbial Nucleotide BLAST, this sequence belongs to tRNA-Pro in the *P. copri* genome. Although the sample was selected based on the presence of 16-20-mers (red sequence in Figure 2F), many registered exRNAs were longer, and we used a *P. copri*-specific 34-mer molecule to test its possible functionality (tRNA sample).

Oligonucleotides *rrl_Pr* (Figure 2G) were found only in *E. coli*-*P. copri* secretomes (Figure 2A) and belong to the end of the prevotella 23S rRNA gene. In the *E. coli* genome, this sequence corresponds to the antisense strand of the *proA* gene in the region of the transcription terminator. We did not register *rrl_Pr* fragments either in the internal transcriptome of *E. coli* or in its secretome (Figure 2A), but inside the cells we found complementary fragments that could be cut from the 3′-UTR of ProA mRNA, where several secondary structures can be formed (shown by arrows in Figure 2G). Taking into account the regulatory potential of some 3′-UTR fragments, we selected both complementary oligonucleotides (*rrl_Pr* and ProA-ter) for experimental assessment.

A total of six samples were selected for further analysis. Fragments *rrl_*2488, *rrl_*2585, and tRNA have sequences present in the genomes of *E. coli* and the competing bacteria, and could appear in joint secretomes from different populations, but almost all RNAs containing discriminator nucleotides belonged to *R. rubrum* or *P. copri*, respectively. The ProA-ter sequence is present in the genomes of *E. coli* and *P. copri*, but can be produced only in *E. coli*, while their complementary sequences–only in *P. copri*. The last sample (*rrs*_−115_) was selected mainly due to its intracellular abundance (Figures 1A, 2A) and also as a potential agent for intraspecific signaling. It is transcribed from an intergenic space and has no sequence homology with the genomes of other model bacteria. Thus, we did not have an opportunity to test the physiological effects of analogs from other genomes.

### Extracellular RNAs Can Alter the Dynamics of *Escherichia coli* Growth

**Figure 3:**
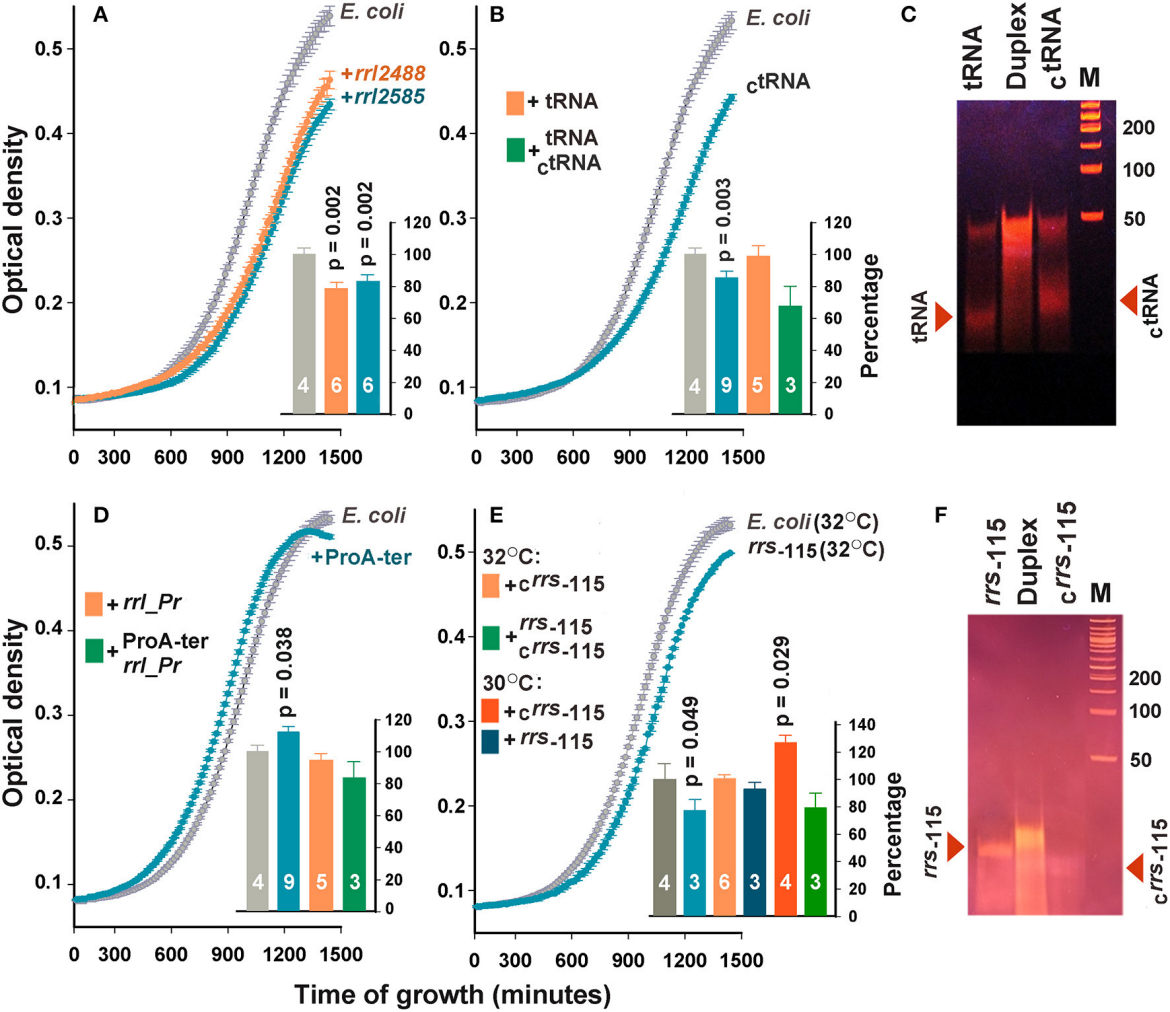
Extracellular RNAs alter the growth dynamics of *E. coli*. Curves in the main plots of **(A,B,D,E)** illustrate the growth rate of *E. coli* in M9 medium at 32°C in the absence (gray plots) and presence (colored plots) of the selected oligonucleotides. The curves obtained in each experiment were averaged over three technical repeats. The number of biological replicates is indicated by white numbers on the columns. Examples of growth curves are shown only for samples with statistically significant effects. Bar plots summarize the results obtained in all experiments of the indicated type. For the samples in plots **(A,B,D)**, the data obtained at 30 and 32°C were pooled and averaged. For the samples *rrs*_−115_ and _C_*rrs*_−115_ **(E)**, the data obtained at different temperatures are shown independently. Error bars represent SEMs calculated for all biological replicates, *p*-values are indicated only in the cases of statistically significant differences from the control samples. Error bars for control samples (light and dark gray bars) show the variability in the growth rate of *E. coli*, estimated from all experiments carried out at 30 **(F)** and 32°C **(A,B,D)**. **(C,F)** illustrate the electrophoretic fractionation profiles of single-stranded oligonucleotides and their complementary duplexes, prepared as described in the section Materials and Methods.

To assess the dependence of the bacterial population on the presence of the selected oligonucleotides, only growth curves were analyzed in this study (Figure 3). Since in many cases the difference between the optical density of the experimental and control samples was time-dependent, we compared the areas under the recorded 24-h growth curves after subtracting the background level. The addition of *rrl_*2488 and *rrl_*2585 to the culturing medium significantly suppressed the growth of *E. coli* (Figure 3A). To our knowledge, this is the first experimental evidence indicating the dependence of *E. coli* growth on the presence of RNAs secreted by a competing bacterium.

No effect was observed for tRNA sample originated from the T*Ψ*C-loop of *P. copri* tRNA-Pro (beige bar in Figure 3B). However, complementary oligonucleotide _C_tRNA demonstrated a well-pronounced inhibitory effect. Upon entering *E. coli* cells, this RNA can base pair with tRNA-Lys promoting its cleavage by double-stranded RNA-specific nucleases. Therefore, we tried to eliminate the inhibitory effect of _C_tRNA by preparing its duplex with tRNA (Figure 3C). Electrophoretic fractionation of single-stranded oligonucleotides revealed a certain portion of duplexes even in their individual samples. This is most likely due to the presence of an inverted repeat in the central part of oligonucleotides. In tRNA, it can form a continuous 10 bp long double-stranded track (5′-GGUUCGAAUC-3′/5′-GGUUCGAAUC-3), and a 12 bp track with two gaps in _C_tRNA (5′-GGaUUCGAAcCU-3′/5′-GGaUUCGAAcCU-3′). In the molten and annealed duplex, single-stranded oligonucleotides were virtually absent (Figure 3C), but the physiological effect was on average stronger compared to single-stranded _C_tRNA, indicating that the tRNA/_C_tRNA duplex may be involved in processes similar to RNA interference. The effect of double-stranded RNA on bacterial growth was previously documented only for microRNAs hsa-miR-515-5p and hsa-miR-1226-5p, but they promoted the cultures of *Fusobacterium nucleatum* and *E. coli*, respectively, rather than suppressed them (Liu et al., 2016). Therefore, the duplex tRNA/_C_tRNA can be used as a model for a detailed study of the suppressor mechanism mediated by secreted RNAs.

The addition of *rrl_Pr* originated from the end of 23S RNA and secreted by *P. copri* also did not change the dynamics of *E. coli* growth (beige bar in Figure 3D), but ProA-ter, which can only be produced by *E. coli*, stimulated the growth of this bacterium (Figure 3D). Thus, it became clear, that *E. coli* endogenous exRNAs can affect the homeostasis of its population exhibiting an opposite effect compared to that registered for *rrl_*2488 and *rrl_*2585. Complementary duplex *rrl_Pr*/ProA-ter, on the contrary, reduced the growth of bacterium to 83.9 ± 10.1%, which statistically significantly differed from the effect rendered by ProA-ter. This can be interpreted as a suppression mediated by RNA secreted by *P. copri*. Considering the faint effect of ProA-ter, we tried to increase it by rising the cultivation temperature from 30°C, previously adjusted to *R. rubrum* growth, to 32°C. However, no significant difference was observed for *rrl_Pr* and ProA-ter, as well as *rrl_*2488, *rrl_*2585, tRNA and _C_tRNA. Thus, the histograms in Figures 3A,B,D represent data averaged over all experiments.

The effect of *rrs*_−115_ sample, selected as a potential agent for intraspecies signaling, on the contrary, turned out to be temperature dependent. We observed a statistically significant decrease in the growth rate at 32°C (Figure 3E), which indicates that endogenous oligonucleotides can exert not only stimulating (Figure 3D), but also inhibitory effects. However, at 30°C the same affect was statistically insignificant. The influence of the complementary oligonucleotide _C_*rrs*_−115_, on the contrary, was well expressed only at 30°C (bar plot in Figure 3E), leading to a 26.1±6.6% increase in growth dynamics. Nearly the same effect from well-formed complementary *rrs*_−115_/_C_*rrs*_−115_ duplex (Figure 3F) and a single stranded *rrs*_−115_, may be even more important, since it indicates that the double-stranded state does not guarantee the highest effect.

### RNAs Secreted by Co-Growing Bacteria Can Enter *E. coli* Cells

Though the observed physiological effects of extracellularly added synthetic oligonucleotides can theoretically be realized both inside and outside the cells, it is much easier to understand and to investigate the mechanisms of action of RNAs capable of penetrating into *E. coli* cells. Therefore, as a final step in this study, we evaluated the ability of two alien oligonucleotides to cross the membrane of *E. coli* cells.

The experiments were conducted using Cy5-labeled synthetic fragments of 23S rRNAs *rrl_2585*, produced by *E. coli* and *R. rubrum* (Figures 2A,E), and *rrl_Pr* transcribed only in *P. copri* (Figures 2A,G), since an identical sequence is found on an antisense strand in the terminator region of the *proA* gene (Figures 2A,G). In order to increase this species specificity of *rrl*_Pr, we extended its sequence by three bases at the 3′-end differing from the sequence in *E. coli* genome. The resulting sequence was 5′-GCACGAATGGTGTAATGAT-Cy5-3′ (5′-GCACGAATGGTGTAATCAC-3′in *E. coli* MG1655). The sequence of *rrl*_2585 was the same as indicated in Figure 2.

**Figure 4:**
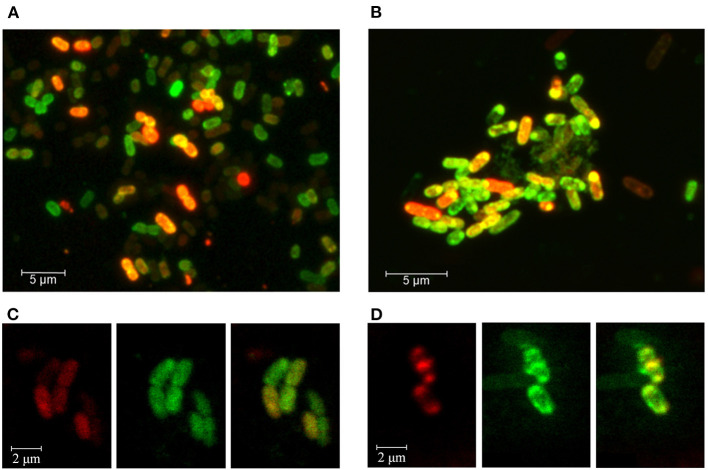
Fluorescence confocal microscopy imaging of *E. coli* cells grown in the presence of Cy5-labeled oligoribonucleotides *rrl*_Pr of *P. copri* **(A,C)** and *rrl*_2585 of *R. rubrum* **(B,D)**. Bacterial cells were cultured for 17 h at 37°C **(A,B)** or 8.5 h at 32°C **(C,D)**. Cell membranes were stained with MitoTracker™ Green FM for 30 min prior to washing-off and microscopy. **(C,D)** are provided as individual fluorescence channels (left and middle blocks) and their superimposition (right blocks).

Bacterial culturing carried out in the presence of modified RNA probes at 37°C, 32°C, or 30°C testified that both molecules can penetrate into the cells, as registered by fluorescence confocal microscopy (Figure 4). The efficiency of MitoTracker™ Green staining done at the end of incubation period was approximately the same in all experiments. Yet, as expected, only part of the cells contained the oligonucleotides tested after 4, 8.5, or 17 h of incubation. Culturing at 37°C provided the highest intensity of Cy5 fluorescence (Figures 4A,B), possibly reflecting the more active physiological state of bacteria at this temperature. However, RNA probes also entered the cells at 32°C (Figures 4C,D), confirming the ability of *rrl_*2585 to affect the growth of *E. coli* (Figure 3A). Although the physiological effect from *rrl_Pr* was much less pronounced (Figure 3D), its intracellular accumulation was registered at approximately the same level, probably indicating a difference in the intracellular functioning of two model oligonucleotides. In any case, it became clear that *E. coli* cells can uptake single-stranded RNA molecules originating from other species and react on their presence in environment even if they are available in a free state. Although the potential mechanisms of their delivery and intracellular functioning remain a topic for further research, it is likely that the observed changes in growth dynamics cannot be explained by simple nutritional effects, since at least four of the eight single-stranded RNAs suppressed the growth of *E. coli*, and only two (ProA-ter and _c_*rrs*_−115_) exhibited notable stimulatory effects.

## Discussion

Although bacteria actively use small regulatory and antisense RNAs for adaptive managing of their transcriptomes, the regulatory potential of secreted oligonucleotides in establishing population homeostasis is still questionable. This is only partly due to the rather short history of research on exRNAs of bacteria and to the many problems associated with their short length and specific processing (Alikina et al., 2018). The main reason seems to reside in the absence of a model approach in formulating an idea of how an RNA-mediated signaling system can work in densely populated and highly heterogeneous natural communities of microorganisms. Based on the knowledge accumulated for eukaryotic microRNAs that massively circulate in the blood and can be delivered to target tissues, it is intuitively expected that RNA interference, involving certain bacterial analogs of Ago proteins and other components of RNA-induced silencing complexes, can function in bacterial communities. In this case, the presence of other bacteria should lead not only to the appearance of new RNAs in the common growth medium, but also to a decrease in the number of some exRNAs. This was indeed the case in our previous study, when the joint secretome of symbiotically grown *E. coli* and *P. bisonicum* was analyzed (Alikina et al., 2018) as well as in the present work, as testified by rigorous differential analysis performed for exRNAs of *E. coli* secreted in the presence of competing bacteria (Figure 1). Thus, the participation of extracellular RNAs in interference is expected, although a decrease in their abundance may occur due to a variety of other regulatory events taking place inside the bacterial cells or in the growth environment.

The main goal of our study was the search for oligonucleotides that can affect the homeostasis of the bacterial population if added extracellularly. Thus, at the first stage we tried to understand which category of secreted RNAs is more promising for identifying functional molecules. Since previously (Alikina et al., 2018) we noticed that the percentage of RNAs mapped to the genome of the co-habiting bacterium *P. bisonicum* is higher in the extracellular fraction compared to the intracellular content, here we evaluated the distribution of *k*-mers, both unique and shared by different genomes, in secretomes depending on the co-grown bacteria. As expected, the percentage of unique *E. coli* exRNAs in the medium of both mixed populations decreased. Strongly opposing the possible assumption that the competing microbes provoke massive secretion of oligonucleotides intended for intraspecific signaling, this also provided indirect evidence in favor of the assumption that in response to the presence of other bacteria *E. coli* secretes oligonucleotides with sequences homologous to their genomes. The species-specific increase in the diversity of homologous RNAs secreted by *E. coli* in mixed population confirms this assumption and is probably one of the most important results of the work.

Based on this view, we considered 28 potential candidates and selected four samples from mixed secretomes for experimental verification. Having discovered a well-pronounced suppression of *E. coli* growth in the presence of *rrl*_2488 and *rrl*_2585 secreted by *R. rubrum*, we for the first time obtained two bacterial sequences, with poorly predictable mode of action due to the collinearity of functional molecules and their potential targets. The suppression mediated by artificial oligonucleotide _C_tRNA, on the contrary, is easier understandable. Being complementary to tRNA-Pro of *P. copri*, _C_tRNA can also base pair with tRNA-Lys of *E. coli*, promoting its cleavage by double-stranded RNA-specific nucleases. In this case, this complementary duplex with weakened base pairing is expected to be less efficient, which was not confirmed, possibly due to dependence of the result from some other molecular or cellular processes. For example, easier penetration of duplex RNAs into bacteria compared to single-stranded oligonucleotides can significantly contribute to the overall biological outcome.

The use of complementary oligonucleotides *rrl_Pr* and ProA-ter provide a chance to evaluate the biological effect of double stranded RNAs, which can be formed by natural molecules produced by two different bacteria. *E. coli* cells actually grew slower in the presence of *rrl_Pr*/ProA-ter duplex (Figure 3D), exemplifying the situation when oligonucleotide secreted by a competing bacterium (*rrl_Pr*) can inhibit the stimulatory effect given by an *E. coli*-specific RNA product (ProA-ter). Given the negative impact of all three duplexes tested (Figures 3B,D,E) it may be prudent to pay attention to secreted oligonucleotides that can form double-stranded RNAs due to their transcription from convergent promoters or processing from stem-loop structures.

Such situation is exemplified by the *rrs*_−115_ sample, which is derived from the leader sequences of all seven 16S rRNAs of *E. coli*, transcribed from two well-characterized promoters located 286 and 178 bp upstream of the 5′-end of rRNA genes. The 5′-end of the *rrs*_−115_ fragment is designated in RegulonDB as a potential transcription start site (TSS) due to its identification in 5′-specific RNA-seq libraries (Santos-Zavaleta et al., 2019; http://regulondb.ccg.unam.mx/index.jsp). However, since there are no promoter-like sequences for this TSS (see, for example PlatProm promoter finder web site http://mathcell.ru/model6.php?l=en, Shavkunov et al., 2009), it is likely that this fragment is a product of RNase processing. Moreover, four potential TSSs were predicted on the opposite strand 16, 34, 48 and 74 bp downstream of the 5′-end of *rrs*_−115_, and transcripts from P_34_ and P_74_ were found in bacterial cells. Therefore, _C_*rrs*_−115_, is also a natural component of the bacterial transcriptome and can be involved in intraspecies signaling.

Thus, using a simple strategy, we identified at least five RNAs (*rrl*_2488, *rrl*_2585, proA-ter, *rrs*_−115_ and _C_*rrs*_−115_) that are produced by bacterial cells and can affect their growth if added extracellularly. Using confocal microscopy, we confirmed the ability of two single-stranded oligonucleotides to penetrate into bacterial cells and anticipate that they retain functionality as it has been demonstrated for eukaryotic microRNAs uptaken by bacterial cells. We also received a large amount of *a priori* unexpected information, such as the low level of intracellular transcriptome contamination from the environment, the functionality of double-stranded RNAs, and the possibility of narrow range temperature dependence for the registered effects. They will definitely come in handy for further research in this area.

## Data Availability Statement

All sequence reads libraries are available at: https://www.ncbi.nlm.nih.gov/bioproject/PRJNA687658.

## Author Contributions

Bacteria were grown and growth curves were obtained by NM and OA. RNAs were isolated and sequenced by OG, KS and OA. Bioinformatic analysis was done by VP and OO, manuscript preparation for publishing was mostly done by OO with a contribution of co-authors. All authors contributed to the article and approved the submitted version.

## Conflict of Interest

The authors declare that the research was conducted in the absence of any commercial or financial relationships that could be construed as a potential conflict of interest.

## Supplementary Material

Supplementary Figure 1 Examples of dynamic curves obtained for *E. coli* (gray plots), *P. copri* (green plots) and *R. rubrum* (red plots) grown in parallel on different media (indicated) at 30°C, in the presence of 1% oxygen, under constant ventilation with CO_2_ (20%) and N_2_ (79%) and with stirring at a speed of 130 RPM. The cultures were inoculated with different dilutions of overnight cultures (1:2000 for *E. coli* and 1:10 for two other bacteria).

Supplementary Figure 2 Examples of growth curves obtained for individually cultivated *E. coli* (gray plots, inoculation ratio 1:4000), *P. copri* (green plots, ratio 1:20) and *R. rubrum* (red plots, ratio 1:10). Dynamic curves for mixed populations *E. coli* (1:4000) + *P. copri* (1:20), *E. coli* (1:4000) + *R. rubrum* (1:10) and *P. copri* (1:20) + *R. rubrum* (1:10) are shown by cyan plots. Magenta filled circles show the expected OD_600_ values for mixed populations if they grow independently during the logarithmic phase when nutrition is not a limiting factor. Vertical dashed lines mark the 8.5-h time point of RNA extraction.

Supplementary Table 1 Genomic distribution of dominant oligonucleotides in the intracellular transcriptome of *E. coli* MG1655.

Supplementary Table 2 Genomic distribution of dominant oligonucleotides in the extracellular RNA fraction of *E. coli* MG1655.

Supplementary Table 3 Genomic distribution of oligonucleotides dominant in the extracellular RNA fraction of mixed population *E. coli* MG1655–*R. rubrum*.

Supplementary Table 4 Genomic distribution of oligonucleotides dominant in the extracellular RNA fraction of mixed population *E. coli* MG1655–*P. copri*.

## References

[B1] Abdullah Z. Schlee M. Roth S. Mraheil M. A. Barchet W. Böttcher J.. (2012). RIG-I detects infection with live Listeria by sensing secreted bacterial nucleic acids. EMBO J. 31, 4153–464. 10.1038/emboj.2012.274 23064150 PMC3492734

[B2] Afgan E. Baker D. van den Beek M. Blankenberg D. Bouvier D. Cech M.. (2016). The Galaxy platform for accessible, reproducible and collaborative biomedical analyses: 2016 update. Nucleic Acids Res. 44, W3–W10. 10.1093/nar/gkw343 27137889 PMC4987906

[B3] Ahmadi Badi S. Bruno S. P. Moshiri A. Tarashi S. Siadat S. D. Masotti A. (2020). Small RNAs in outer membrane vesicles and their function in host-microbe interactions. Front. Microbiol. 11:1209. 10.3389/fmicb.2020.01209 32670219 PMC7327240

[B4] Alikina O. V. Glazunova O. A. Bykov A. A. Kiselev S. S. Tutukina M. N. Shavkunov K. S.. (2018). A cohabiting bacterium alters the spectrum of short RNAs secreted by Escherichia coli. FEMS Microbiol. Lett. 365:fny262. 10.1093/femsle/fny262 30376063

[B5] Antipov S. S. Tutukina M. N. Preobrazhenskaya E. V. Kondrashov F. A. Patrushev M. V. Toshchakov S. V.. (2017). The nucleoid protein Dps binds genomic DNA of Escherichia coli in a non-random manner. PLoS ONE 12:e0182800. 10.1371/journal.pone.0182800 28800583 PMC5553809

[B6] Appling D. R. (2008). Software Review of Prism 5. J. Am. Chem. Soc. 130:6056. 10.1021/ja801998j

[B7] Arumugam M. Raes J. Pelletier E. Paslier D. Yamada T. Mende D. R.. (2011). Enterotypes of the human gut microbiome. Nature 473, 174–80. 10.1038/nature09944 21508958 PMC3728647

[B8] Beatty M. Guduric-Fuchs J. Brown E. Bridgett S. Chakravarthy U. Hogg R.. (2014). Small RNAs from plants, bacteria and fungi within the order Hypocreales are ubiquitous in human plasma. BMC Genomics 15:933. 10.1186/1471-2164-15-933 25344700 PMC4230795

[B9] Bechhofer D. H. Deutscher M. P. (2019). Bacterial ribonucleases and their roles in RNA metabolism. Crit. Rev. Biochem. Mol. Biol. 54, 242–300. 10.1080/10409238.2019.1651816 31464530 PMC6776250

[B10] Blattner F. R. Plunkett G. 3rd Bloch C. A. Perna N. T. Burland V. Riley M.. (1997). The complete genome sequence of *Escherichia coli* K-12. Science 277, 1453–1462. 10.1126/science.277.5331.1453 9278503

[B11] Blenkiron C. Simonov D. Muthukaruppan A. Tsai P. Dauros P. Green S.. (2016). Uropathogenic Escherichia coli releases extracellular vesicles that are associated with RNA. PLoS ONE 11:e0160440. 10.1371/journal.pone.0160440 27500956 PMC4976981

[B12] Bloch S. Wegrzyn A. Wegrzyn G. Nejman-Faleńczyk B. (2017). Small and smaller-sRNAs and MicroRNAs in the regulation of toxin gene expression in prokaryotic cells: a mini-review. Toxins 9:181. 10.3390/toxins9060181 28556797 PMC5488031

[B13] Bykov A. A. Shavkunov K. S. Panyukov V. V. Ozoline O. N. (2016). Bacterial nucleoid protein Dps binds structured RNA molecules. Mat. Biolog. Bioinform. 11, 311–322. 10.17537/2016.11.311

[B14] Carver T. Thomson N. Bleasby A. Berriman M. Parkhill J. (2009). DNAPlotter: circular and linear interactive genome visualization. Bioinformatics 25, 119–20. 10.1093/bioinformatics/btn578 18990721 PMC2612626

[B15] Castel S. E. Martienssen R. A. (2013). RNA interference in the nucleus: roles for small RNAs in transcription, epigenetics and beyond. Nat. Rev. Genet. 14, 100–12. 10.1038/nrg3355 23329111 PMC4205957

[B16] Chao Y. Li L. Girodat D. Förstner K. U. Said N. Corcoran C.. (2017). *In vivo* cleavage map illuminates the central role of RNase E in coding and non-coding RNA pathways. Mol. Cell. 65, 39–51. 10.1016/j.molcel.2016.11.002 28061332 PMC5222698

[B17] Chen J. Conn S. (2017). Canonical mRNA is the exception, rather than the rule. Genome Biol. 18:133. 10.1186/s13059-017-1268-1 28687088 PMC5501951

[B18] Cheng L. Sharples R. A. Scicluna B. J. Hill A. F. (2014). Exosomes provide a protective and enriched source of miRNA for biomarker profiling compared to intracellular and cell-free blood. J. Extracell. Vesicles 3:23743. 10.3402/jev.v3.23743 24683445 PMC3968297

[B19] d'Adda di Fagagna F. (2014). A direct role for small non-coding RNAs in DNA damage response. Trends Cell Biol. 24, 171–18. 10.1016/j.tcb.2013.09.008 24156824

[B20] Dalmasso G. Nguyen H. T. Yan Y. Laroui H. Charania M. A. Ayyadurai S.. (2011). Microbiota modulate host gene expression via microRNAs. PLoS ONE 6:e19293. 10.1371/journal.pone.0019293 21559394 PMC3084815

[B21] Davis B. M. Waldor M. K. (2007). RNase E-dependent processing stabilizes MicX, a Vibrio cholerae sRNA. Mol. Microbiol. 65, 373–85. 10.1111/j.1365-2958.2007.05796.x 17590231 PMC1976385

[B22] Diebel K. W. Zhou K. Clarke A. B. Bemis L. T. (2016). Beyond the ribosome: extra-translational functions of tRNA fragments. Biomark. Insights 11, 1–8. 10.4137/BMI.S35904 26843810 PMC4734663

[B23] Eigenbrod T. Pelka K. Latz E. Kreikemeyer B. Dalpke A. H. (2015). TLR8 senses bacterial RNA in human monocytes and plays a nonredundant role for recognition of *Streptococcus pyogenes*. J. Immunol. 195, 1092–1099. 10.4049/jimmunol.1403173 26101323

[B24] Eisenhardt K. M. H. Reuscher C. M. Klug G. (2018). PcrX, an sRNA derived from the 3′- UTR of the Rhodobacter sphaeroides puf operon modulates expression of puf genes encoding proteins of the bacterial photosynthetic apparatus. Mol. Microbiol. 110, 325–334. 10.1111/mmi.14076 29995316

[B25] Forde B. M. O'Toole P. W. (2013). Next-generation sequencing technologies and their impact on microbial genomics. Brief. Funct. Genomics 12, 440–53. 10.1093/bfgp/els062 23314033

[B26] Fritz J. V. Heintz-Buschart A. Ghosal A. Wampach L. Etheridge A. Galas D.. (2016). Sources and functions of extracellular small RNAs in human circulation. Annu. Rev. Nutr. 36, 301–36. 10.1146/annurev-nutr-071715-050711 27215587 PMC5479634

[B27] Geyer M. Pelka K. Latz E. (2015). Synergistic activation of Toll-like receptor 8 by two RNA degradation products. Nat. Struct. Mol. Biol. 22, 99–101. 10.1038/nsmb.2967 25650902

[B28] Ghosal A. Upadhyaya B. B. Fritz J. V. Heintz-Buschart A. Desai M. S. Yusuf D.. (2015). The extracellular RNA complement of *Escherichia coli*. MicrobiologyOpen 4, 252–66. 10.1002/mbo3.235 25611733 PMC4398507

[B29] Glinge C. Clauss S. Boddum K. Jabbari R. Jabbari J. Risgaard B.. (2017). Stability of circulating blood-based MicroRNAs - pre-analytic methodological considerations. PLoS ONE 12:e0167969. 10.1371/journal.pone.0167969 28151938 PMC5289450

[B30] Heil F. Hemmi H. Hochrein H. Ampenberger F. Kirschning C. Akira S.. (2004). Species-specific recognition of single-stranded RNA via toll-like receptor 7 and 8. Science 303, 1526–159. 10.1126/science.1093620 14976262

[B31] Hoyos M. Huber M. Förstner K. U. Papenfort K. (2020). Gene autoregulation by 3′ UTR-derived bacterial small RNAs. eLife 9:e58836. 10.7554/eLife.58836 32744240 PMC7398697

[B32] Hungate R. E. (1969). A roll tube method for cultivation of strict anaerobes. Methods Microbiol. 3, 117–132. 10.1016/S0580-9517(08)70503-8

[B33] Jung S. von Thülen T. Laukemper V. Pigisch S. Hangel D. Wagner H.. (2015). A single naturally occurring 2'-O-methylation converts a TLR7- and TLR8-activating RNA into a TLR8-specific ligand. PLoS ONE 10:e0120498. 10.1371/journal.pone.0120498 25785446 PMC4364935

[B34] Kaiser I. Oelze J. (1980). Grow and adaptation to phototrophic conditions of Rhodospirillum rubrum and Rhodopseudomonas sphaeroides at different temperatures. Arch. Microbiol. 126, 187–194. 10.1007/BF00511226

[B35] Kaiser S. Rimbach K. Eigenbrod T. Dalpke A. H. Helm M. (2014). A modified dinucleotide motif specifies tRNA recognition by TLR7. RNA 20, 1351–135. 10.1261/rna.044024.113 25051971 PMC4138318

[B36] Kang S. M. Choi J. W. Lee Y. Hong S. H. Lee H. J. (2013). Identification of microRNA-size, small RNAs in Escherichia coli. Curr. Microbiol. 67, 609–13. 10.1007/s00284-013-0411-9 23783561

[B37] Koeppen K. Hampton T. H. Jarek M. Scharfe M. Gerber S. A. Mielcarz D. W.. (2016). A novel mechanism of host-pathogen interaction through sRNA in bacterial outer membrane vesicles. PLoS Pathog. 12:e1005672. 10.1371/journal.ppat.1005672 27295279 PMC4905634

[B38] Krüger A. Oldenburg M. Chebrolu C. Beisser D. Kolter J. Sigmund A. M.. (2015). Human TLR8 senses UR/URR motifs in bacterial and mitochondrial RNA. EMBO Rep. 16, 1656–1663. 10.15252/embr.201540861 26545385 PMC4687425

[B39] Kukurba K. R. Montgomery S. B. (2015). RNA sequencing and analysis. Cold Spring Harb. Protoc. 2015, 951–969. 10.1101/pdb.top084970 25870306 PMC4863231

[B40] Kulp A. J. Sun B. Ai T. Manning A. J. Orench-Rivera N. Schmid A. K.. (2015). Genome-wide assessment of outer membrane vesicle production in *Escherichia coli*. PLoS ONE 10:e0139200. 10.1371/journal.pone.0139200 26406465 PMC4583269

[B41] Lalaouna D. Carrier M. C. Massé E. (2015a). Every little piece counts: the many faces of tRNA transcripts. Transcription 6, 74–7. 10.1080/21541264.2015.1093064 26595434 PMC4802806

[B42] Lalaouna D. Carrier M. C. Semsey S. Brouard J. S. Wang J. Wade J. T.. (2015b). A 3′ external transcribed spacer in a tRNA transcript acts as a sponge for small RNAs to prevent transcriptional noise. Mol. Cell. 58, 393–405. 10.1016/j.molcel.2015.03.013 25891076

[B43] Lee H. J. Hong S. H. (2012). Analysis of microRNA-size, small RNAs in Streptococcus mutans by deep sequencing. FEMS Microbiol. Lett. 326, 131–16. 10.1111/j.1574-6968.2011.02441.x 22092283

[B44] Leighton L. J. Bredy T. W. (2018). Functional interplay between small non-coding RNAs and RNA modification in the brain. Non-coding RNA 4:15. 10.3390/ncrna4020015 29880782 PMC6027130

[B45] Levanova A. Poranen M. M. (2018). RNA interference as a prospective tool for the control of human viral infections. Front. Microbiol. 9:2151. 10.3389/fmicb.2018.02151 30254624 PMC6141738

[B46] Liu S. da Cunha A. P. Rezende R. M. Cialic R. Wei Z. Bry L.. (2016). The host shapes the gut microbiota via fecal MicroRNA. Cell Host Microbe. 19, 32–43. 10.1016/j.chom.2015.12.005 26764595 PMC4847146

[B47] Malabirade A. Habier J. Heintz-Buschart A. May P. Godet J. Halder R.. (2018). The RNA complement of outer membrane vesicles from salmonella enterica serovar typhimurium under distinct culture conditions. Front. Microbiol. 9:2015. 10.3389/fmicb.2018.02015 30214435 PMC6125333

[B48] Mall C. Rocke D. M. Durbin-Johnson B. Weiss R. H. (2013). Stability of miRNA in human urine supports its biomarker potential. Biomark. Med. 7, 623–31. 10.2217/bmm.13.44 23905899 PMC3885156

[B49] Mills K. H. (2011). TLR-dependent T cell activation in autoimmunity. Nat. Rev. Immunol. 11, 807–22. 10.1038/nri3095 22094985

[B50] Miyakoshi M. Matera G. Maki K. Sone Y. Vogel J. (2019). Functional expansion of a TCA cycle operon mRNA by a 3′ end-derived small RNA. Nucleic Acids Res. 47, 2075–2088. 10.1093/nar/gky1243 30541135 PMC6393394

[B51] Miyoshi T. Ito K. Murakami R. Uchiumi T. (2016). Structural basis for the recognition of guide RNA and target DNA heteroduplex by Argonaute. Nat. Commun. 7:11846. 10.1038/ncomms11846 27325485 PMC4919518

[B52] Munk A. C. Copeland A. Lucas S. Lapidus A. del Rio T. D. Barry K.. (2011). Complete genome sequence of *Rhodospirillum rubrum* type strain (S1). Stand. Genomic Sci. 4, 293–302. 10.4056/sigs.1804360 21886856 PMC3156396

[B53] Nakanishi K. (2016). Anatomy of RISC: how do small RNAs and chaperones activate Argonaute proteins? Wiley Interdiscip. Rev. RNA. 7, 637–60. 10.1002/wrna.1356 27184117 PMC5084781

[B54] Nozawa R. S. Boteva L. Soares D. C. Naughton C. Dun A. R. Buckle A.. (2017). SAF-A regulates interphase chromosome structure through oligomerization with chromatin-associated RNAs. Cell 169, 1214–1227.e18. 10.1016/j.cell.2017.05.029 28622508 PMC5473940

[B55] Obbard D. J. Gordon K. H. Buck A. H. Jiggins F. M. (2009). The evolution of RNAi as a defence against viruses and transposable elements. Philos. Trans. R. Soc. Lond. B. Biol. Sci. 364, 99–115. 10.1098/rstb.2008.0168 18926973 PMC2592633

[B56] O'Donoghue E. J. Krachler A. M. (2016). Mechanisms of outer membrane vesicle entry into host cells. Cell. Microbiol. 18, 1508–1517. 10.1111/cmi.12655 27529760 PMC5091637

[B57] Panyukov V. V. Kiselev S. S. Alikina O. V. Nazipova N. N. Ozoline O. N. (2017). Short unique sequences in bacterial genomes as strain- and species-specific signatures. Math. Biol. Bioinf. 12, 547–58. 10.17537/2017.12.547

[B58] Panyukov V. V. Kiselev S. S. Ozoline O. N. (2020). Unique k-mers as strain-specific barcodes for phylogenetic analysis and natural microbiome profiling. Int. J. Mol. Sci. 21:944. 10.3390/ijms21030944 32023871 PMC7037511

[B59] Panyukov V. V. Kiselev S. S. Shavkunov K. S. Masulis I. S. Ozoline O. N. (2013). Mixed promoter islands as genomic regions with specific structural and functional properties. Math. Biol. Bioinf. 8, 432–448. 10.17537/2013.8.432

[B60] Papenfort K. Said N. Welsink T. Lucchini S. Hinton J. C. D. Vogel J. (2009). Specific and pleiotropic patterns of mRNA regulation by ArcZ, a conserved, Hfq-dependent small RNA. Mol. Microbiol. 74, 139–158. 10.1111/j.1365-2958.2009.06857.x 19732340

[B61] Pavankumar A. R. Ayyappasamy S. P. Sankaran K. (2012). Small RNA fragments in complex culture media cause alterations in protein profiles of three species of bacteria. BioTechniques 52, 167–72. 10.2144/000113819 22401549

[B62] Rosace D. López J. Blanco S. (2020). Emerging roles of novel small non-coding regulatory RNAs in immunity and cancer. RNA Biol. 17, 1196–1213. 10.1080/15476286.2020.1737442 32186461 PMC7549716

[B63] Santos-Zavaleta A. Salgado H. Gama-Castro S. Sánchez-Pérez M. Gómez-Romero L. Ledezma-Tejeida D.. (2019). RegulonDB v 10.5: tackling challenges to unify classic and high throughput knowledge of gene regulation in *E. coli* K-12. Nucleic Acids Res. 47, D212–D220. 10.1093/nar/gky1077 30395280 PMC6324031

[B64] Shavkunov K. S. Masulis I. S. Tutukina M. N. Deev A. A. Ozoline O. N. (2009). Gains and unexpected lessons from genome-scale promoter mapping. Nucleic Acids Res. 37, 4919–4931. 10.1093/nar/gkp490 19528070 PMC2731890

[B65] Swiatowy W. Jagodzińśki P. P. (2018). Molecules derived from tRNA and snoRNA: entering the degradome pool. Biomed. Pharmacother. 108, 36–42. 10.1016/j.biopha.2018.09.017 30216797

[B66] Wang C. Chao Y. Matera G. Gao Q. Vogel J. (2020). The conserved 3′ UTR-derived small RNA NarS mediates mRNA crossregulation during nitrate respiration. Nucleic Acids Res. 48, 2126–2143. 10.1093/nar/gkz1168 31863581 PMC7038943

[B67] Wiegand C. Heusser P. Klinger C. Cysarz D. Büssing A. Ostermann T.. (2018). Stress-associated changes in salivary microRNAs can be detected in response to the Trier Social Stress Test: an exploratory study. Sci. Rep. 8:7112. 10.1038/s41598-018-25554-x 29740073 PMC5940676

[B68] Willkomm S. Zander A. Gust A. Grohmann D. (2015). A prokaryotic twist on argonaute function. Life (Basel). 5, 538–53. 10.3390/life5010538 25692904 PMC4390867

[B69] Wolfe R. S. (1971). Microbial formation of methane. Adv. Microb. Physiol. 6, 107–46. 10.1016/S0065-2911(08)60068-5 4950179

[B70] Yau T. O. Tang C. M. Harriss E. K. Dickins B. Polytarchou C. (2019). Faecal microRNAs as a non-invasive tool in the diagnosis of colonic adenomas and colorectal cancer: a meta-analysis. Sci. Rep. 9:9491. 10.1038/s41598-019-45570-9 31263200 PMC6603164

