# Supplementary figures and images for: Suppression of Escherichia coli Growth Dynamics via RNAs Secreted by Competing Bacteria
Source: Front Mol Biosci. 2021 Apr 15;8:609979. doi: 10.3389/fmolb.2021.609979 (PMC8082180; doi:10.3389/fmolb.2021.609979)

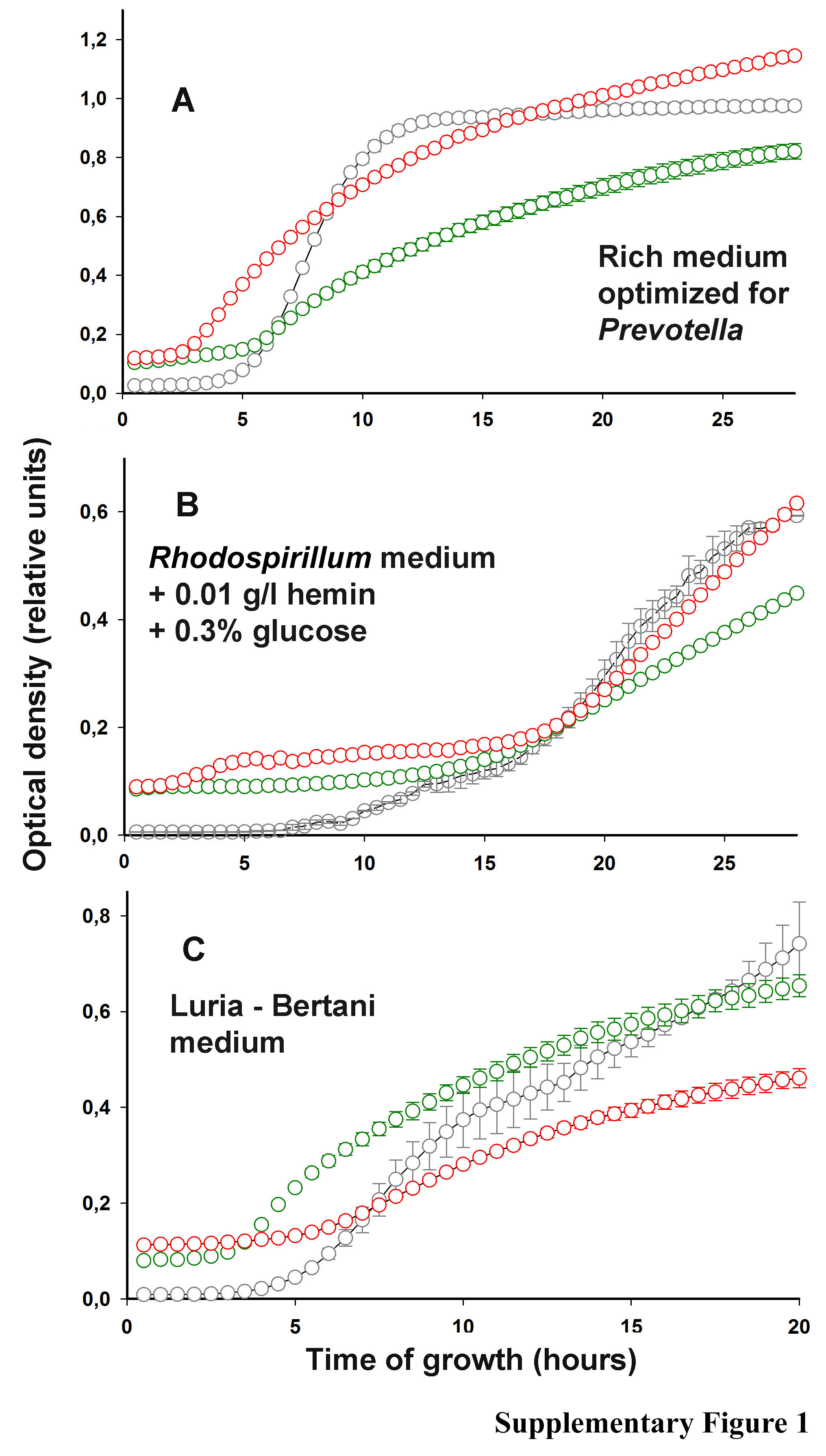

Supplement: Supplementary Figure 1 — Examples of dynamic curves obtained for E. coli (gray plots), P. copri (green plots) and R. rubrum (red plots) grown in parallel on different media (indicated) at 30°C, in the presence of 1% oxygen, under constant ventilation with CO2 (20%) and N2 (79%) and with stirring at a speed of 130 RPM. The cultures were inoculated with different dilutions of overnight cultures (1:2000 for E. coli and 1:10 for two other bacteria). [file Image_1.TIF]

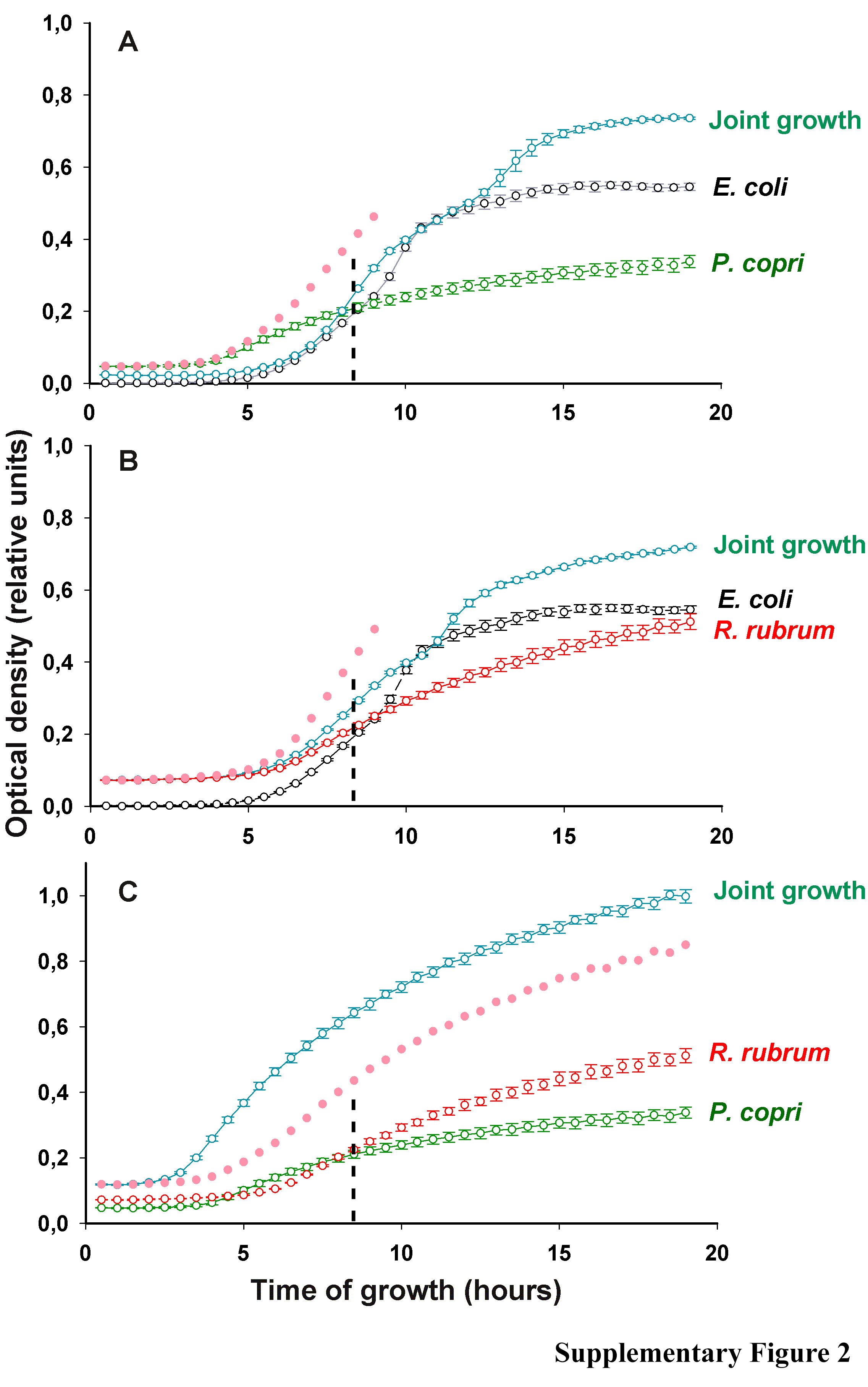

Supplement: Supplementary Figure 2 — Examples of growth curves obtained for individually cultivated E. coli (gray plots, inoculation ratio 1:4000), P. copri (green plots, ratio 1:20) and R. rubrum (red plots, ratio 1:10). Dynamic curves for mixed populations E. coli (1:4000) + P. copri (1:20), E. coli (1:4000) + R. rubrum (1:10) and P. copri (1:20) + R. rubrum (1:10) are shown by cyan plots. Magenta filled circles show the expected OD600 values for mixed populations if they grow independently during the logarithmic phase when nutrition is not a limiting factor. Vertical dashed lines mark the 8.5-h time point of RNA extraction. [file Image_2.TIF]
