# Supplementary material for: Suppression of Escherichia coli Growth Dynamics via RNAs Secreted by Competing Bacteria
Source: Front Mol Biosci. 2021 Apr 15;8:609979. doi: 10.3389/fmolb.2021.609979 (PMC8082180; doi:10.3389/fmolb.2021.609979)
Supplement: Supplementary Table 1 — Genomic distribution of dominant oligonucleotides in the intracellular transcriptome of E. coli MG1655. [file Table_1.DOCX]

**Supplementary Table 1. Genomic distribution of dominant oligonucleotides in the intracellular transcriptome of *E. coli* MG1655**

|  | Position of the 5’-end | Str. | Number  of  reads in the peak | Associated genes | | | | TSP | Type of genomic  loci |
| --- | --- | --- | --- | --- | --- | --- | --- | --- | --- |
|  |  |  |  | Gene name | 5'-end  position | 3'-end  position | Str. |  |  |
| 1 | 12085 | + | 468 | *dnaK* | 12163 | 14079 | + | **12048** | mRNA fragments |
| 2 | 122923 | + | 747 | *tp2* | 122697 | 122857 | - |  | Intergenic |
|  |  |  |  | *aceE* | 123017 | 125680 | + |  |  |
| 3 | 122969 | + | 814 | *aceE* | 123017 | 125680 | + | **122969** | mRNA 5'-end |
| 4 | 223656 | + | 2260 | *gmhB* | 222833 | 223408 | + | **223593** | Intergenic,  ***rrsH*_-115_** |
|  |  |  |  | *rrsH* | 223771 | 225312 | + |  |  |
| 5 | 225346 | + | 806 | *rrsH* | 223771 | 225312 | + |  | Intergenic |
|  |  |  |  | *ileV* | 225381 | 225457 | + |  |  |
| 6 | 225381 | + | 303 | *ileV* | 225381 | 225457 | + | 225381 | tRNA 5'-end |
| 7 | 225500 | + | 558 | *alaV* | 225500 | 225575 | + |  | tRNA 5'-end |
| 8 | 228230 | + | 313 | *rrlH* | 225759 | 228662 | + |  | 23S RNA fragments |
| 9 | 228645 | + | 465 | *rrlH* | 225759 | 228662 | + |  | 23S RNA 3'-end |
| 10 | 228928 | + | 981 | *aspU* | 228928 | 229004 | + |  | tRNA 5'-end |
| 11 | 236931 | + | 981 | *aspV* | 236931 | 237007 | + |  | tRNA 5'-end |
| 12 | 237010 | + | 243 | *aspV* | 236931 | 237007 | + |  | Intergenic |
|  |  |  |  | *yafT* | 237335 | 238120 | + |  |  |
| 13 | 262871 | + | 179 | *thrW* | 262871 | 262946 | + | **262837** | tRNA 5'-end |
| 14 | 407175 | + | 186 | *yaiA* | 406979 | 407170 | + |  | Intergenic |
|  |  |  |  | *aroM* | 407428 | 408105 | + |  |  |
| 15 | 696740 | - | 854 | *metU* | 696664 | 696740 | - |  | tRNA 5'-end |
| 16 | 697047 | - | 1137 | *leuW* | 696963 | 697047 | - |  | tRNA 5'-end |
| 17 | 697133 | - | 854 | *metT* | 697057 | 697133 | - |  | tRNA 5'-end |
| 18 | 780554 | + | 510 | *lysT* | 780554 | 780629 | + | **780512** | tRNA 5'-end |
| 19 | 780765 | + | 200 | *valT* | 780765 | 780840 | + |  | tRNA 5'-end |
| 20 | 780843 | + | 510 | *lysW* | 780843 | 780918 | + |  | tRNA 5'-end |
| 21 | 781068 | + | 200 | *valZ* | 781068 | 781143 | + |  | tRNA 5'-end |
| 22 | 781147 | + | 510 | *lysY* | 781147 | 781222 | + |  | tRNA 5'-end |
| 23 | 781369 | + | 510 | *lysZ* | 781369 |  | + |  | tRNA 5'-end |
| 24 | 781577 | + | 510 | *lysQ* | 781577 | 781652 | + |  | tRNA 5'-end |
| 25 | 925971 | - | 229 | *serW* | 925884 | 925971 | - | **926008** | tRNA 5'-end |
| 26 | 1097652 | - | 229 | *serX* | 1097565 | 1097652 | - | **1097679** | tRNA 5'-end |
| 27 | 1165991 | + | 186 | *ycfP* | 1165143 | 1165685 | + | 1165991 | Intergenic |
|  |  |  |  | *ndh* | 1166085 | 1167389 | + |  |  |
| 28 | 1169034 | + | 476 | *bhsA* | 1169073 | 1169330 | + | **1169022** | 5'-UTR |
| 29 | 1258738 | + | 347 | *ychH* | 1258791 | 1259069 | + | **1258738** | mRNA fragments |
| 30 | 1269330 | + | 231 | *rdlA* | 1269323 | 1269389 | + | **1269323** | sRNA fragments |
| 31 | 1269864 | + | 273 | *rdlB* | 1269858 | 1269923 | + | **1269858** | sRNA fragments |
|  | 1269865 | + | 206 |  |  |  |  |  |  |
| 32 | 1270399 | + | 186 | *rdlC* | 1270393 | 1270460 | + | **1270393** | sRNA fragments |
| 33 | 1398760 | - | 195 | *uspE* | 1397672 | 1398622 | - |  | Intergenic |
|  |  |  |  | *fnr* | 1398774 | 1399526 | - |  |  |
| 34 | 1741201 | + | 194 | *ydhC* | 1739911 | 1741122 | + | **1741201** | mRNA 5'-end |
|  |  |  |  | *cfa* | 1741413 | 1742561 | + |  |  |
| 35 | 1746435 | + | 724 | *valV* | 1746435 | 1746511 | + | **1746415** | tRNA 5'-end |
| 36 | 1746516 | + | 468 | *valW* | 1746516 | 1746592 | + |  | tRNA 5'-end |
| 37 | 1757383 | + | 266 | *pykF* | 1755698 | 1757110 | + | **1757383** | mRNA 5'-end |
|  |  |  |  | *lpp* | 1757421 | 1757657 | + |  |  |
| 38 | 1923157 | + | 746 | *RyeA* | 1923066 | 1923337 | + | 1923093 | sRNA fragments |
| 39 | 1923168 | - | 275 | *sdsR* | 1923104 | 1923207 | - | **1923204** | sRNA fragments |
| 40 | 1992117 | - | 421 | *glyW* | 1992042 | 1992117 | - | **1992183** | tRNA 5'-end |
| 41 | 2044549 | + | 179 | *asnT* | 2044549 | 2044624 | + | **2044538** | tRNA 5'-end |
| 42 | 2058051 | - | 270 | *asnW* | 2058027 | 2058102 | - | 2058113 | tRNA3'-part |
| 43 | 2059851 | + | 179 | *asnU* | 2059851 | 2059926 | + | **2059842** | tRNA 5'-end |
| 44 | 2062260 | + | 179 | *asnV* | 2062260 | 2062335 | + | **2062249** | tRNA 5'-end |
| 45 | 2520931 | + | 239 | *valU* | 2520931 | 2521006 | + | **2520922** | tRNA 5'-end |
| 46 | 2521051 | + | 200 | *valX* | 2521051 | 2521126 | + |  | tRNA 5'-end |
| 47 | 2521173 | + | 200 | *valY* | 2521173 | 2521248 | + |  | tRNA 5'-end |
| 48 | 2521253 | + | 510 | *lysV* | 2521253 | 2521328 | + |  | tRNA 5'-end |
| 49 | 2726082 | - | 592 | *rrfG* | 2726069 | 2726188 | - |  | 5S RNA 3'-end |
| 50 | 2726298 | - | 471 | *rrlG* | 2726281 | 2729184 | - |  | 23S RNA 3'-end |
| 51 | 2726713 | - | 323 | *rrlG* | 2726281 | 2729184 | - |  | 23S RNA fragments |
| 52 | 2729582 | - | 1483 | *gltW* | 2729369 | 2729444 | - | 2729678 | Intergenic, |
|  | 2729606 | - | 203 | *rrsG* | 2729616 | 2731157 | - |  |  |
| 53 | 2731272 | - | 2257 | *rrsG* | 2729616 | 2731157 | - |  | Intergenic  ***rrsG*_-115_** |
|  |  |  |  | *clpB* | 2731600 | 2734173 | - |  |  |
| 54 | 2755593 | + | 263 | *ssrA* | 2755593 | 2755955 | + | **2755586** | TmRNA 5'-end |
| 55 | 3055974 | + | 337 | *zapA* | 3055612 | 3055941 | + | **3055974** | sRNA 5'-end |
|  |  |  |  | *ssrS* | 3055983 | 3056165 | + |  |  |
| 56 | 3056851 | +/- | 182 | *sibC* | 3056851 | 3056991 | + | **3056851** | sRNA 5'-end |
| 57 | 3318375 | - | 337 | *metY* | 3318213 | 3318289 | - | **3318375** | Intergenic |
|  |  |  |  | *argG* | 3318637 | 3319980 | + |  |  |
| 58 | 3350642 | + | 699 | *arcZ* | 3350577 | 3350697 | + | **3350577** | sRNA 3'-part |
| 59 | 3423436 | - | 877 | *rrfF* | 3423423 | 3423542 | - |  | 5S RNA 3'-end |
| 60 | 3423655 | - | 397 | *thrV* | 3423580 | 3423655 | - |  | tRNA 5'-end |
| 61 | 3423681 | - | 592 | *rrfD* | 3423668 | 3423787 | - |  | 5S RNA 3'-end |
| 62 | 3423897 | - | 472 | *rrlD* | 3423880 | 3426783 | - |  | 23S RNA 3'-end |
| 63 | 3424312 | - | 323 | *rrlD* | 3423880 | 3426783 | - |  | 23S RNA fragments |
| 64 | 3427033 | - | 575 | *alaU* | 3426958 | 3427033 | - |  | tRNA 5'-end |
| 65 | 3427187 | - | 787 | *ileU* | 3427076 | 3427152 | - |  | Intergenic |
|  | 3427221 | - | 203 | *rrsD* | 3427221 | 3428762 | - |  |  |
| 66 | 3428877 | - | 2257 | *rrsD* | 3427221 | 3428762 | - |  | Intergenic,  ***rrsD*_-115_** |
|  |  |  |  | *yrdA* | 3429236 | 3429790 | + |  |  |
| 67 | 3630929 | - | 287 | *yhiY* | 3630665 | 3630778 | - | 3630929 | Intergenic |
|  |  |  |  | *yhiJ* | 3630968 | 3632590 | - |  |  |
| 68 | 3639984 | + | 227 | *uspB* | 3639385 | 3639720 | - | **3639984** | mRNA 5'-end |
|  |  |  |  | *uspA* | 3640111 | 3640545 | + |  |  |
| 69 | 3655961 | - | 427 | *yhiD* | 3655255 | 3655902 | - | 3655972 | Intergenic |
|  |  |  |  | *hdeB* | 3655966 | 3656292 | - |  |  |
| 70 | 3836197 | + | 995 | *yicJ* | 3834547 | 3835929 | - |  | 5'-end of tRNA precursor |
|  | 3836198 |  | 409 | *selC* | 3836222 | 3836316 | + | 3836197 |  |
| 71 | 3933640 | +  + | 212 | *rbsD* | 3933351 | 3933770 | + |  | mRNA fragmentss |
|  | 3933645 |  | 508 |  |  |  |  |  |  |
| 72 | 3941693 | + | 2260 | *yieP* | 3940635 | 3941327 | - | **3941634** | Intergenic  ***rrsC*_-115_** |
|  |  |  |  | *rrsC* | 3941808 | 3943349 | + |  |  |
| 73 | 3943383 | + | 1096 | *rrsC* | 3941808 | 3943349 | + |  | Intergenic |
|  |  |  |  | *gltU* | 3943435 | 3943510 | + |  |  |
| 74 | 3946175 | + | 313 | *rrlC* | 3943704 | 3946607 | + |  | 23S RNA fragments |
| 75 | 3946590 | + | 465 | *rrlC* | 3943704 | 3946607 | + |  | 23S RNA fragments |
| 76 | 3946632 | + | 304 | *rrlC* | 3943704 | 3946607 | + |  | Intergenic |
|  |  |  |  | *rrfC* | 3946700 | 3946819 | + |  |  |
| 77 | 3946872 | + | 981 | *aspT* | 3946872 | 3946948 | + |  | tRNA 5'-end |
| 78 | 3946957 | + | 542 | *trpT* | 3946957 | 3947032 | + |  | tRNA 5'-end |
| 79 | 3982509 | + | 1008 | *hisR* | 3982509 | 3982585 | + |  | tRNA 5'-end |
| 80 | 3982606 | + | 789 | *leuT* | 3982606 | 3982692 | + |  | tRNA 5'-end |
| 81 | 4035416 | + | 2260 | *hemG* | 4034608 | 4035153 | + |  | Intergenic  **rrsA**_-115_ |
|  |  |  |  | *rrsA* | 4035531 | 4037072 | + | **4035357** |  |
| 82 | 4037106 | + | 806 | *rrsA* | 4035531 | 4037072 | + |  | Intergenic |
|  |  |  |  | *ileT* | 4037141 | 4037217 | + |  |  |
| 83 | 4037141 | + | 308 | *ileT* | 4037141 | 4037217 | + | **4037141** | tRNA 5'-end |
| 84 | 4037260 | + | 558 | *alaT* | 4037260 | 4037335 | + |  | tRNA 5'-end |
| 85 | 4039991 | + | 313 | *rrlA* | 4037519 | 4040423 | + |  | 23 S RNA fragments |
| 86 | 4040406 | + | 465 | *rrlA* | 4037519 | 4040423 | + |  | 23S RNA 3'-end |
|  |  |  |  | *rrfA* | 4040517 | 4040636 | + |  |  |
| 87 | 4040517 | + | 175 | *rrfA* | 4040517 | 4040636 | + |  | 5S RNA 5'-end |
| 88 | 4049899 | + | 271 | *spf* | 4049899 | 4050007 | + | **4049899** | sRNA 5'-end |
| 89 | 4166544 | + | 2260 | *murI* | 4165428 | 4166285 | + | **4166487** | Intergenic  ***rrsB*_-115_** |
|  |  |  |  | *rrsB* | 4166659 | 4168200 | + |  |  |
| 90 | 4168234 | + | 1506 | *rrsB* | 4166659 | 4168200 | + |  | Intergenic |
|  |  |  |  | *gltT* | 4168372 | 4168447 | + |  |  |
| 91 | 4171112 | + | 313 | *rrlB* | 4168641 | 4171544 | *+* |  | 23 S RNA fragments |
| 92 | 4171527 | + | 465 | *rrlB* | 4168641 | 4171544 | + |  | 23S RNA 3'-end |
| 93 | 4171569 | + | 304 | *rrlB* | 4168641 | 4171544 | + |  | Intergenic |
|  |  |  |  | *rrfB* | 4171637 | 4171756 | + |  |  |
| 94 | 4171637 | + | 175 | *rrfB* | 4171637 | 4171756 | + |  | 5S RNA 5'-end |
| 95 | 4175388 | + | 269 | *thrU* | 4175388 | 4175463 | + | **4175381** | tRNA 5'-end |
| 96 | 4175673 | + | 558 | *glyT* | 4175673 | 4175747 | + |  | tRNA 5'-end |
| 97 | 4175754 | + | 339 | *thrT* | 4175754 | 4175829 | + |  | tRNA 5'-end |
| 98 | 4208032 | + | 2260 | *purH* | 4205943 | 4207532 | - | **4207971** | Intergenic  ***rrsE*_-115_** |
|  |  |  |  | *rrsE* | 4208147 | 4209688 | + |  |  |
| 99 | 4209722 | + | 1505 | *rrsE* | 4208147 | 4209688 | + |  | Intergenic |
|  |  |  |  | *gltV* | 4209774 | 4209849 | + |  |  |
| 100 | 4212514 | + | 313 | *rrlE* | 4210043 | 4212946 |  |  | 23 S RNA fragments |
| 101 | 4212929 | + | 465 | *rrlE* | 4210043 | 4212946 | + |  | 23S RNA 3'-end |
| 102 | 4213040 | + | 175 | *rrfE* | 4213040 | 4213159 | + |  | 5S RNA 5'-end |
| 103 | 4392360 | + | 665 | *glyV* | 4392360 | 4392435 | + | **4392310** | tRNA 5'-end |
| 104 | 4392472 | + | 665 | *glyX* | 4392472 | 4392547 | + |  | tRNA 5'-end |
| 105 | 4392583 | + | 665 | *glyY* | 4392583 | 4392658 | + |  | tRNA 5'-end |
| 106 | 4416764 | - | 1824 | *bsmA* | 4416441 | 4416770 | - | **4416867** | mRNA 5'-end |
| 107 | 4439821 | + | 460 | *ytfK* | 4439587 | 4439793 | + |  | Intergenic |
|  |  |  |  | *ytfL* | 4439872 | 4441215 | - |  |  |
| 108 | 4543673 | + | 375 | *fimA* | 4543115 | 4543663 | + | 4543604 | Intergenic |
|  |  |  |  | *fimI* | 4543728 | 4544267 | + |  |  |
| 109 | 4606165 | - | 805 | *leuV* | 4606079 | 4606165 | - |  | tRNA 5'-end |
| 110 | 4606286 | - | 839 | *leuP* | 4606200 | 4606286 | - |  | tRNA 5'-end |
| 111 | 4606401 | - | 839 | *leuQ* | 4606315 | 4606401 | - | **4606434** | tRNA 5'-end |

The Table shows the positional coordinates in the *E. coli* genome for all the peak maxima registered in the Eco_in experiment (Table 1 in the main text) with at least 150 sequence reads. “+/-” in the third column (Str. = strand) indicates that along with main oligonucleotides derived from the “+” strand, there are few complementary transcripts from the “-” strand. Genome annotation was taken from RegulonDB (http://regulondb.ccg.unam.mx). The search for transcription start points (TSP) was done within 100 bp upstream from the 5’-end of the detected oligonucleotides using the PlatProm promoter finder (http://mathcell.ru/model6.php?l=en, Shavkunov et al. 2009). Their genomic coordinates are in bold if the corresponding promoters are indicated in RegulonDB.
