# Supplementary material for: Suppression of Escherichia coli Growth Dynamics via RNAs Secreted by Competing Bacteria
Source: Front Mol Biosci. 2021 Apr 15;8:609979. doi: 10.3389/fmolb.2021.609979 (PMC8082180; doi:10.3389/fmolb.2021.609979)
Supplement: Supplementary Table 2 — Genomic distribution of dominant oligonucleotides in the extracellular RNA fraction of E. coli MG1655. [file Table_2.DOCX]

**Supplementary Table 2. Genomic distribution of dominant oligonucleotides in the extracellular RNA fraction of *E. coli* MG1655**

|  | Position of the 5’-end | Str. | Number  of  reads in the peak | Associated genes | | | | TSP | Type of genomic  loci |
| --- | --- | --- | --- | --- | --- | --- | --- | --- | --- |
|  |  |  |  | Gene name | 5'-end  position | 3'-end  position | Str. |  |  |
| 1 | 32232 | + | 238 | *carB* | 30817 | 34038 | + |  | mRNA fragments |
| 2 | 69547 | - | 33/2 | *araB* | 68348 | 70048 | - |  | mRNA fragments |
| 3 | 192704 | + | 21/0 | *pyrH* | 191855 | 192580 | + | 192646 | Intergenic |
|  |  |  |  | *frr* | 192872 | 193429 | + |  |  |
| 4 | 202154 | + | 31 | *fabZ* | 202101 | 202556 | + | 202136 | mRNA fragments |
| 5 | **223656** | + | 21 | *gmhB* | 222833 | 223408 | + |  | Intergenic  ***rrsH***_-115_ |
|  |  |  |  | *rrsH* | 223771 | 225312 | + | **223593** |  |
| 6 | 225759 | + | 43 | *rrlH* | 225759 | 228662 | + | 225719 | 23S RNA 5'-end |
| 7 | 230112 | +/- | 42 | *yafC* | 229967 | 230881 | - |  | Antisense RNAs |
| 8 | 251076 | + | 21/1 | *dinB* | 250898 | 251953 | + |  | mRNA fragments |
| 9 | 347315 | - | 23/21 | *prpR* | 346857 | 348443 | - |  | mRNA fragments |
| 10 | 360276 | + | 36 | *cynX* | 359992 | 361146 | + |  | mRNA fragments |
| 11 | 418317 | + | 35 | *phoR* | 417889 | 419184 | + |  | mRNA fragments |
| 12 | 461638 | - | 91 | *hupB* | 461451 | 461723 | + | 461666 | Antisense RNAs |
| 13 | 499830 | + | 28/23 | *aes* | 499014 | 499973 | - | 499832 | Antisense RNAss |
| 14 | 499831 | + | 22 |  |  |  |  |  |  |
| 15 | 572562 | - | 57/39 | *ybcN* | 572466 | 572921 | + |  | Antisense RNAs |
| 16 | 789928 | + | 26/0 | *galK* | 788831 | 789979 | - |  | Antisense RNAs |
| 17 | 799556 | + | 31/0/0 | *pgl* | 798586 | 799581 | + |  | mRNA 3'-end |
|  | 799557 | + | 21/1/0 |  |  |  |  |  |  |
| 18 | 836050 | - | 26 | *ybiB* | 835248 | 836210 | + |  | Antisense RNAs |
| 19 | 837243 | - | 32/0 | *hcxB* | 836351 | 837436 | + | 837268 | Antisense RNAs |
| 20 | 851867 | + | 54 | *opgE* | 851014 | 852597 | - |  | Antisense RNAs |
| 21 | 858566 | - | 26/9 | *ybiU* | 857796 | 859061 | - |  | mRNA fragments |
|  | 858567 | - | 54/1 |  |  |  |  |  |  |
| 22 | 911196 | - | 22 | *hcr* | 911182 | 912150 | - |  | mRNA 3'-end |
| 23 | 1035762 | - | 26 | *hyaC* | 1035066 | 1035773 | + |  | Antisense to mRNA junction |
|  |  |  |  | *hyaD* | 1035770 | 1036357 | + |  |  |
| 24 | 1149693 | + | 44/1 | *fabH* | 1148759 | 1149712 | + |  | mRNA fragments |
| 25 | 1302440 | + | 53/0 | *oppA* | 1301182 | 1302813 | + | 1302384 | mRNA fragments |
| 26 | 1305928 | + | 32/8 | *oppF* | 1305764 | 1306768 | + |  | mRNA fragments |
| 27 | 1406579 | +/- | 49/1 | *dgcM* | 1406563 | 1407795 | - |  | Antisense RNAs |
| 28 | 1583771 | - | 24/2 | *safA* | 1583762 | 1583959 | - | PI | mRNA 3'-end |
| 29 | 1591034 | + | 37 | *hipA* | 1590854 | 1592176 | - | 1590992 | Antisense RNAs |
| 30 | 1680068 | + | 24/3/3 | *ydgI* | 1679557 | 1680939 | + |  | mRNA fragments |
| 31 | 1694930 | - | 25 | *uidA* | 1694260 | 1696071 | - | 1694989 | mRNA fragments |
| 32 | 1711244 | - | 65/59 | *rsxE* | 1710828 | 1711523 | + |  | Antisense RNAs |
| 33 | 1748124 | - | 38/1 | *ydhS* | 1747131 | 1748735 | + | 1748154 | Antisense RNAs |
| 34 | 1861401 | - | 31/2 | *ydjL* | 1860256 | 1861332 | - | 1861413 | Intergenic |
|  |  |  |  | *yeaC* | 1861702 | 1861974 | - |  |  |
| 35 | 1896004 | - | 40 | *pabB* | 1894805 | 1896166 | + |  | Antisense RNAs |
| 36 | 1918172 | - | 26/0 | *yebT* | 1917510 | 1920143 | + | 1918196 | Antisense RNAs |
| 37 | 1931024 | + | 42/30/30 | *purT* | 1930881 | 1932059 | + |  | mRNA fragments |
| 38 | 1972599 | - | 22/0/0 | *tar* | 1971030 | 1972691 | - | 1972673 | mRNA fragments |
| 39 | 1996121 | - | 86/0 | *sdiA* | 1996110 | 1996832 | - | 1996161 | mRNA 3'-end |
| 40 | 2003390 | + | 21 | *fliC* | 2002110 | 2003606 | - |  | Antisense RNAs |
| 41 | 2118510 | - | 28 | *wcaK* | 2117124 | 2118404 | - |  | Intergenic  (Rep-element) |
|  |  |  |  | *wzxC* | 2118680 | 2120158 | - |  |  |
| 42 | 2160237 | + | 31/14 | *mdtC* | 2158386 | 2161463 | + | 2160171 | mRNA fragments |
| 43 | 2299478 | - | 55 | *napH* | 2298715 | 2299578 | - |  | mRNA fragments |
| 44 | 2309398 | - | 65/0 | *ada* | 2309341 | 2310405 | - | 2309454 | mRNA fragments |
| 45 | 2337451 | + | 27 | *gyrA* | 2336793 | 2339420 | - |  | Antisense RNAs |
| 46 | 2455375 | - | 34/28 | *yfcV* | 2455083 | 2455646 | - |  | mRNA fragments |
| 47 | 2592890 | - | 25/0/24 | *ypfN* | 2592762 | 2592962 | + |  | Antisense RNAs |
| 48 | 2631171 | - | 83/82 | *guaA* | 2630958 | 2632535 | - |  | mRNA fragments |
| 49 | 2645862 | - | 24/0/19 | *pbpC* | 2645013 | 2647325 | - | 2645863 | mRNA fragments |
| 50 | 2669421 | - | 55/3 | *hcaE* | 2669032 | 2670393 | + |  | Antisense RNAs |
| 51 | 2674086 | + | 32/29/29 | *yphB* | 2673816 | 2674688 | - |  | Antisense RNAs |
|  | 2674087 | + | 92/0 |  |  |  |  |  |  |
| 52 | 2675086 | - | 161/3/3 | *yphC* | 2674700 | 2675761 | - |  | mRNA fragments |
| 53 | 2682157 | - | 40/0 | *yphG* | 2679464 | 2682745 | - |  | mRNA fragments |
| 54 | 2714833 | + | 27/0/0 | *yfiE* | 2714439 | 2715320 | - |  | Antisense RNAs |
| 55 | 2737291 | - | 124/110 | *raiA* | 2737154 | 2737495 | + |  | Antisense RNAs |
|  |  |  |  | *raiZ* | 2737381 | 2737542 | + |  | mRNA leader |
| 56 | 2781826 | + | 71 | *ypjA* | 2778146 | 2782726 | - | 2781794 | Antisense RNAs |
| 57 | 2822502 | + | 37/0 | *pncC* | 2823849 | 2824346 | - |  | Antisense RNAs |
| 58 | 2845132 | + | 62 | *hycE* | 2844762 | 2846471 | - | 2845038 | Antisense RNAs |
| 59 | 2881263 | + | 33/0 | *casC* | 2881051 | 2882142 | - | 2881261 | Antisense RNAs |
| 60 | 2909501 | - | 30 | *pyrG* | 2908029 | 2909666 | - | 2909573 | mRNA fragments |
| 61 | 2958044 | - | 28/7 | *ptrA* | 2955996 | 2958884 | - | 2958041 | mRNA fragments |
| 62 | 2991781 | + | 32 | *ygeG* | 2991268 | 2991759 | + | PI | Intergenic |
|  |  |  |  | *ygeH* | 2992094 | 2993470 | + |  |  |
| 63 | 3034407 | + | 23/0 | *lysS* | 3033657 | 3035174 | - | 3034345 | Antisense RNAs |
| 64 | 3046934 | - | 34 | *gcvP* | 3046168 | 3049041 | - |  | mRNA fragments |
| 65 | 3047984 | - | 104/0 | *gcvP* | 3046168 | 3049041 | - | 3048006 | mRNA fragments |
| 66 | 3105214 | +/- | 21 | *mltC* | 3104433 | 3105512 | + |  | mRNA fragments |
| 67 | 3108810 | - | 28/27 | *speC* | 3107020 | 3109155 | - |  | mRNA fragments |
| 68 | 3111478 | - | 30/0 | *yghE* | 3111128 | 3111988 | - |  | mRNA fragments |
| 69 | 3135469 | + | 25 | *pitB* | 3134872 | 3136371 | - |  | Antisense RNAs |
| 70 | 3192106 | - | 238 | *glgS* | 3191739 | 3191939 | - | MPI | Intergenic |
|  |  |  |  | *yqiJ* | 3192208 | 3192837 | + |  |  |
| 71 | 3265937 | + | 34/31 | *tdcB* | 3265039 | 3266028 | - | 3265935 | Antisense RNAs |
| 72 | 3289146 | + | 30/1/2 | *yraJ* | 3288814 | 3291330 | + | 3289137 | mRNA fragments |
| 73 | 3291383 | + | 26/0/0 | *yraK* | 3291341 | 3292432 | + |  | mRNA fragments |
| 74 | 3326865 | - | 22 | *ftsH* | 3325001 | 3326935 | - | 3326909 | mRNA fragments |
| 75 | 3344580 | + | 24 | *lptB* | 3343944 | 3344669 | + |  | mRNA fragments |
| 76 | 3345351 | + | 57/35 | *rpoN* | 3344717 | 3346150 | + |  | mRNA fragments |
| 77 | 3407743 | + | 24/2/2 | *panF* | 3407607 | 3409058 | + |  | mRNA fragments |
| 78 | 3435536 | - | 21 | *rsmB* | 3435207 | 3436496 | + | 3435559 | Antisense RNAs |
| 79 | 3450951 | + | 43/4 | *rplB* | 3450543 | 3451364 | - | 3450950 | Antisense RNAs |
| 80 | 3467240 | + | 36 | *chiA* | 3467160 | 3469853 | - | 3467199 | Antisense RNAs |
| 81 | 3513124 | - | 45/1/1 | *trpS* | 3512634 | 3513638 | - |  | mRNA fragments |
| 82 | 3533981 | - | 25/19 | *pck* | 3532818 | 3534440 | + |  | Antisense RNAs |
| 83 | 3543448 | - | 30 | *rpnA* | 3543167 | 3544045 | + |  | Antisense RNAs |
| 84 | 3641126 | + | 28/0/0 | *dtpB* | 3640862 | 3642331 | + | 3641088 | mRNA fragments |
| 85 | 3770242 | - | 67/0 | *yibH* | 3770243 | 3771379 | - |  | 3'-UTR |
| 86 | 3836960 | + | 21/12 | *setC* | 3836953 | 3838137 | + | PI | mRNA 5'-end |
| 87 | 3882828 | - | 43 | *dnaA* | 3882326 | 3883729 | - |  | mRNA fragments |
| 88 | 3918588 | - | 32 | *atpA* | 3918316 | 3919857 | - |  | mRNA fragments |
| 89 | 3922927 | - | 53/44 | *atpI* | 3922060 | 3922440 | - | PI | Intergenic |
|  | 3922928 | - | 106 | *rsmG* | 3923057 | 3923680 | - |  |  |
| 90 | **3941693** | + | 21 | *yieP* | 3940635 | 3941327 | - |  | Intergenic  ***rrsC*_-115_** |
|  |  |  |  | *rrsC* | 3941808 | 3943349 | + | **3941634** |  |
| 91 | 3943704 | + | 43 | *rrlC* | 3943704 | 3946607 | + | 3943622 | 23S RNA 5'-end |
| 92 | **4035416** | + | 21 | *hemG* | 4034608 | 4035153 | + |  | Intergenic  ***rrsA*_-115_** |
|  |  |  |  | *rrsA* | 4035531 | 4037072 | + | **4035357** |  |
| 93 | 4037519 | + | 43 | *rrlA* | 4037519 | 4040423 | + |  | 23S RNA 5'-end |
| 94 | **4166544** | + | 21 | *murI* | 4165428 | 4166285 | + |  | Intergenic  ***rrsB*_-115_** |
|  |  |  |  | *rrsB* | 4166659 | 4168200 | + | **4166487** |  |
| 95 | 4168641 | + | 43 | *rrlB* | 4168641 | 4171544 | + | 4168559 | 23S RNA 5'-end |
| 96 | 4198774 | + | 30/12 | *hemE* | 4197716 | 4198780 | + | 4198697 | mRNA 3'-end |
| 97 | 4200467 | -  - | 20/0/0 | *hupA* | 4200281 | 4200553 | + | 4200496 | Antisense RNAs |
|  | 4200468 |  | 71 |  |  |  |  |  |  |
| 98 | **4208032** | + | 21 | *purH* | 4205943 | 4207532 | - |  | Intergenic  ***rrsE*_-115_** |
|  |  |  |  | *rrsE* | 4208147 | 4209688 | + | **4207971** |  |
| 99 | 4210043 | + | 43 | *rrlE* | 4210043 | 4212946 | + | 4209971 | 23S RNA 5'-end |
| 100 | 4236699 | - | 23/0 | *yjbF* | 4236262 | 4236900 | + |  | Antisense RNAs |
| 101 | 4337030 | - | 24/10 | *adiC* | 4335694 | 4337031 | - | 4337029 | mRNA 3'-end |
| 102 | 4347120 | - | 22/0 | *fumB* | 4345680 | 4347326 | - |  | mRNA fragments |
| 103 | 4447721 | - | 27 | *tamB* | 4444112 | 4447891 | + | 4447752 | Antisense RNAs |
| 104 | 4578142 | + | 35 | *mcrB* | 4577958 | 4579337 | - |  | Antisense RNAs |

The Table shows the positional coordinates in the *E. coli* genome for all the peaks found in the combined set of RNAs secreted by *E. coli* in experiments Eco_out_1 and Eco_out_2 (Table 1 in the main text), containing more than 20 sequence reads in the peak maxima. The positions of the peaks overlapping with Supplementary Table 1 are shown in bold. “+/-” in the third column (Str. = strand) indicates that along with main oligonucleotides derived from the “+” strand, there is at least one complementary transcript from the “-” strand. The fourth column shows the total number of reads in the peaks before deletion of reads corresponding to the genomes of model bacteria used for co-growth with *E. coli* (black), as well as after removal of oligonucleotides that map to the genomes of *R. rubrum* (red) or *P. copri* (green). Genome annotation was taken from RegulonDB (http://regulondb.ccg.unam.mx). The search for transcription start points (TSP) was done within 100 bp upstream from the 5’-end of the detected oligonucleotides using the PlatProm promoter finder (http://mathcell.ru/model6.php?l=en, Shavkunov et al. 2009). Their genomic coordinates are in bold if the corresponding promoters are indicated in RegulonDB. “PI” in this column means the presence of a *Promoter Island* in the region with multiple sigma-70 promoters on both strands, from which the detected oligonucleotides can be transcribed (described in Shavkunov et al. 2009). “MPI” means the presence of a Mixed Promoter Island in the region containing multiple promoters with different sigma-specificity (described in Panyukov et al. 2013).
