# Supplementary material for: Suppression of Escherichia coli Growth Dynamics via RNAs Secreted by Competing Bacteria
Source: Front Mol Biosci. 2021 Apr 15;8:609979. doi: 10.3389/fmolb.2021.609979 (PMC8082180; doi:10.3389/fmolb.2021.609979)
Supplement: Supplementary Table 3 — Genomic distribution of oligonucleotides dominant in the extracellular RNA fraction of mixed population E. coli MG1655–R. rubrum. [file Table_3.DOCX]

**Supplementary Table 3. Genomic distribution of oligonucleotides dominant in the extracellular RNA fraction of mixed population *E. coli* MG1655 – *R. rubrum***

|  | Position of the 5’-end | Str. | Number  of  reads in the peak | Associated genes | | | | TSP | Type of genomic  loci |
| --- | --- | --- | --- | --- | --- | --- | --- | --- | --- |
|  |  |  |  | Gene name | 5'-end  position | 3'-end  position | Str. |  |  |
| 1 | **32232** | + | 217/217 | *carB* | 30817 | 34038 | + |  | mRNA fragments |
| 2 | 88622 | + | 21/17 | *cra* | 88028 | 89032 | + |  | mRNA fragments |
| 3 | 91918 | - | 18/18 | *ftsI* | 91413 | 93179 | + |  | Antisense RNAs |
| 4 | **192704** | + | 19/0 | *pyrH* | 191855 | 192580 | + | 192646 | Intergenic |
|  |  |  |  | *frr* | 192872 | 193429 | + |  |  |
| 5 | **202154** | + | 31/31 | *fabZ* | 202101 | 202556 | + | 202136 | mRNA fragments |
| 6 | 224543 | + | 87/0 | *rrsH* | 223771 | 225312 | + | 224454 | 16S RNA fragments |
| 7 | **251076** | + | 26/3 | *dinB* | 250898 | 251953 | + |  | mRNA fragments |
| 8 | 261518 | + | 37/7 | *proA* | 261503 | 262756 | + |  | tRNA fragments |
| 9 | 323248 | - | 17/16 | *ykgF* | 322338 | 323765 | + |  | Antisense RNAs |
| 10 | 331439 | - | 21/21 | *betT* | 329463 | 331496 | + |  | Antisense RNAs |
| 11 | **347315** | - | 18/18 | *prpR* | 346857 | 348443 | - |  | mRNA fragments |
| 12 | **418317** | + | 16/16 | *phoR* | 417889 | 419184 | + |  | mRNA fragments |
| 13 | **461637** | - | 26/1 | *hupB* | 461451 | 461723 | + | 461666 | Antisense RNAs |
| 14 | 461638 | - | 47/47 |  |  |  |  |  |  |
| 15 | 474268 | + | 25/19 | *amtB* | 472966 | 474252 | + |  | Intergenic |
|  |  |  |  | *tesB* | 474301 | 475161 | - |  |  |
| 16 | **499830** | + | 20/17 | *aes* | 499014 | 499973 | - | 499832 | Antisense RNAs |
| 17 | **499831** | + | 19/19 |  |  |  |  |  |  |
| 18 | **572564** | - | 30/30 | *ybcN* | 572466 | 572921 | + |  | Antisense RNAs |
| 19 | **789928** | + | 20/20 | *galK* | 788831 | 789979 | - |  | Antisense RNAs |
| 20 | **799556** | + | 29/0 | *pgl* | 798586 | 799581 | + |  | mRNA 3'-end |
|  | **799557** | + | 18/0 |  |  |  |  |  |  |
| 21 | **836050** | - | 25/25 | *ybiB* | 835248 | 836210 | + |  | Antisense RNAs |
| 22 | **837243** | - | 22/0 | *hcxB* | 836351 | 837436 | + | 837268 | Antisense RNAs |
| 23 | **851867** | + | 44/44 | *opgE* | 851014 | 852597 | - |  | Antisense RNAs |
| 24 | **858566** | - | 33/33 | *ybiU* | 857796 | 859061 | - |  | mRNA fragments |
|  | **858567** | - | 36/36 |  |  |  |  |  |  |
| 25 | 911906 | - | 18/7 | *hcr* | 911182 | 912150 | - | 911940 | mRNA fragments |
| 26 | 958192 | + | 33/33 | *serC* | 957653 | 958741 | + |  | mRNA fragments |
| 27 | 960848 | - | 19/19 | *ycaL* | 960264 | 961028 | + |  | Antisense RNAs |
| 28 | **1035762** | - | 17/17 | *hyaC* | 1035066 | 1035773 | + |  | Antisense to mRNAs junction |
|  |  |  |  | *hyaD* | 1035770 | 1036357 | + |  |  |
| 29 | 1112710 | + | 25/19 | *b1049* | 1110863 | 1113406 | + |  | mRNA fragments |
| 30 | 1125263 | - | 24/0 | *mdtH* | 1124118 | 1125326 | - |  | mRNA fragments |
| 31 | **1302440** | + | 30/30 | *oppA* | 1301182 | 1302813 | + | 1302384 | mRNA fragments |
| 32 | **1305928** | + | 19/19 | *oppF* | 1305764 | 1306768 | + |  | mRNA fragments |
| 33 | **1406579** | + | 25/0 | *dgcM* | 1406563 | 1407795 | - |  | Antisense RNAs |
| 34 | 1506475 | - | 20/16 | *sutR* | 1506172 | 1506708 | + |  | Antisense RNAs |
| 35 | **1583771** | - | 18/18 | *safA* | 1583762 | 1583959 | - | PI | mRNA 3'-end |
| 36 | 1606438 | - | 16/16 | *lsrF* | 1606100 | 1606975 | + |  | Antisense RNAs |
| 37 | **1694930** | - | 37/37 | *uidA* | 1694260 | 1696071 | - | 1694989 | mRNA fragments |
| 38 | **1711244** | - | 78/78 | *rsxE* | 1710828 | 1711523 | + |  | Antisense RNAs |
| 39 | **1748124** | - | 16/16 | *ydhS* | 1747131 | 1748735 | + | 1748154 | Antisense RNAs |
| 40 | **1896004** | - | 37/0 | *pabB* | 1894805 | 1896166 | + |  | Antisense RNAs |
| 41 | **1931024** | + | 28/21 | *purT* | 1930881 | 1932059 | + |  | mRNA fragments |
| 42 | **1996122** | - | 89/89 | *sdiA* | 1996110 | 1996832 | - | 1996161 | mRNA 3'-end |
| 43 | **2118510** | **-** | 38/38 | *wcaK* | 2117124 | 2118404 | - |  | Intergenic,  Rep-element |
|  |  |  |  | *wzxC* | 2118680 | 2120158 | - |  |  |
| 44 | 2150397 | - | 21/21 | *yegI* | 2149039 | 2150985 | - | 2150461 | mRNA fragments |
| 45 | 2152536 | + | 18/0 | *yegL* | 2152469 | 2153128 | - |  | Antisense RNAs |
| 46 | **2160237** | + | 35/17 | *mdtC* | 2158386 | 2161463 | + | 2160171 | mRNA fragments |
| 47 | 2223637 | - | 16/16 | *dld* | 2222185 | 2223900 | + |  | Antisense RNAs |
| 48 | **2299478** | - | 33/33 | *napH* | 2298715 | 2299578 | - |  | mRNA fragments |
| 49 | **2309398** | - | 62/62 | *ada* | 2309341 | 2310405 | - | 2309454 | mRNA fragments |
| 50 | **2337451** | + | 25/25 | *gyrA* | 2336793 | 2339420 | - |  | Antisense RNAs |
| 51 | 2424624 | - | 25/2 | *hisM* | 2424517 | 2425233 | - |  | mRNA fragments |
| 52 | **2592892** | - | 28/0 | *ypfN* | 2592762 | 2592962 | + |  | Antisense RNAs |
| 53 | **2631172** | - | 44/44 | *guaA* | 2630958 | 2632535 | - |  | mRNA fragments |
| 54 | **2645863** | - | 18/0 | *pbpC* | 2645013 | 2647325 | - | 2645863 | mRNA fragments |
| 55 | **2669423** | - | 52/52 | *hcaE* | 2669032 | 2670393 | + |  | Antisense RNAs |
| 56 | **2674086** | + | 29/27 | *yphB* | 2673816 | 2674688 | - |  | Antisense RNAs |
|  | **2674087** | + | 81/81 |  |  |  |  |  |  |
| 57 | **2675086** | - | 150/3 | *yphC* | 2674700 | 2675761 | - |  | mRNA fragments |
| 58 | **2682157** | - | 44/0 | *yphG* | 2679464 | 2682745 | - |  | mRNA fragments |
| 59 | 2730385 | - | 84/0 | *rrsG* | 2729616 | 2731157 | - | 2730474 | 16S RNA fragments |
| 60 | **2737293** | - | 109/109 | *raiA* | 2737154 | 2737495 | + |  | Antisense RNAs |
|  |  |  |  | *raiZ* | 2737381 | 2737542 | + |  | mRNA leader |
| 61 | **2781826** | + | 39/39 | *ypjA* | 2778146 | 2782726 | - | 2781794 | Antisense RNAs |
| 62 | **2822502** | + | 42/0 | *recX* | 2822139 | 2822639 | - |  | Antisense RNAs |
| 63 | **2845132** | + | 42/42 | *hycE* | 2844762 | 2846471 | - | 2845038 | Antisense RNAs |
| 64 | 2887892 | + | 23/0 | *cysH* | 2887578 | 2888312 | - | 2887793 | Antisense RNAs |
| 65 | **2909501** | - | 40/40 | *pyrG* | 2908029 | 2909666 | - | 2909573 | mRNA fragments |
| 66 | 2936065 | + | 16/1 | *fucI* | 2935584 | 2937359 | + |  | mRNA fragments |
| 67 | **2991781** | + | 22/22 | *ygeG* | 2991268 | 2991759 | + | PI  **2991876** | Intergenic |
|  |  |  |  | *ygeH* | 2992094 | 2993470 | + |  |  |
| 68 | **3046934** | - | 29/29 | *gcvP* | 3046168 | 3049041 | - |  | mRNA fragments |
| 69 | **3047984** | - | 66/0 | *gcvP* | 3046168 | 3049041 | - | 3048006 | mRNA fragments |
| 70 | **3111478** | - | 31/31 | *yghE* | 3111128 | 3111988 | - |  | mRNA fragments |
| 71 | **3192106** | - | 217/217 | *glgS* | 3191739 | 3191939 | - | MPI | Intergenic |
|  |  |  |  | *yqiJ* | 3192208 | 3192837 | + |  |  |
| 72 | 3210793 | + | 26/26 | *rpsU* | 3210781 | 3210996 | + | **3210735** | mRNA fragments |
| 73 | 3237794 | + | 18/18 | *ygjR* | 3237311 | 3238297 | + | 3237792 | mRNA fragments |
| 74 | **3265937** | + | 22/22 | *tdcB* | 3265039 | 3266028 | - | 3265935 | Antisense RNAs |
| 75 | **3289146** | + | 31/0 | *yraJ* | 3288814 | 3291330 | + | 3289137 | mRNA fragments |
| 76 | **3344580** | + | 24/24 | *lptB* | 3343944 | 3344669 | + |  | mRNA fragments |
| 77 | **3345351** | + | 46/33 | *rpoN* | 3344717 | 3346150 | + |  | mRNA fragments |
| 78 | 3427990 | - | 84/0 | *rrsD* | 3427221 | 3428762 | - | 3428079 | 16S RNA fragments |
| 79 | **3450951** | + | 53/1 | *rplB* | 3450543 | 3451364 | - | 3450950 | Antisense RNAs |
| 80 | 3455765 | - | 18/18 | *gspC* | 3455578 | 3456393 | + | PI | Antisense RNAs |
| 81 | **3467240** | + | 30/30 | *chiA* | 3467160 | 3469853 | - | 3467199 | Antisense RNAs |
| 82 | **3513124** | - | 36/0 | *trpS* | 3512634 | 3513638 | - |  | mRNA fragments |
| 83 | **3533981** | - | 31/31 | *pck* | 3532818 | 3534440 | + |  | Antisense RNAs |
| 84 | **3543448** | - | 20/20 | *rpnA* | 3543167 | 3544045 | + |  | Antisense RNAs |
| 85 | 3562866 | + | 26/0 | *glpD* | 3562013 | 3563518 | + |  | mRNA fragments |
| 86 | **3641126** | + | 29/1 | *dtpB* | 3640862 | 3642331 | + | 3641088 | mRNA fragments |
| 87 | **3770242** | - | 68/0 | *yibH* | 3770243 | 3771379 | - |  | 3'-UTR |
| 88 | 3800488 | - | 20/20 | *waaY* | 3800267 | 3800965 | - | 3800488 | mRNA fragments |
| 89 | **3882828** | - | 48/48 | *dnaA* | 3882326 | 3883729 | - |  | mRNA fragments |
| 90 | **3918588** | - | 33/33 | *atpA* | 3918316 | 3919857 | - |  | mRNA fragments |
| 91 | **3922928** | - | 145/145 | *atpI* | 3922060 | 3922440 | - | PI | Intergenic |
|  |  |  |  | *rsmG* | 3923057 | 3923680 | - |  |  |
| 92 | 3942580 | + | 89/0 | *rrsC* | 3941808 | 3943349 | + | 3942491 | 16S RNA fragments |
| 93 | 4036303 | + | 89/0 | *rrsA* | 4035531 | 4037072 | + | 4036214 | 16S RNA fragments |
| 94 | 4104684 | - | 35/17 | *cpxA* | 4103602 | 4104975 | - |  | mRNA fragments |
| 95 | 4142794 | - | 28/28 | *frwC* | 4142530 | 4143609 | + |  | Antisense RNAs |
| 96 | 4167431 | + | 89/0 | *rrsB* | 4166659 | 4168200 | + | 4167342 | 16S RNA fragments |
| 97 | **4200467** | - | 26/0 | *hupA* | 4200281 | 4200553 | + | 4200496 | Antisense RNAs |
|  | **4200468** | - | 47/47 |  |  |  |  |  |  |
| 98 | 4208919 | + | 89/0 | *rrsE* | 4208147 | 4209688 | + | 4208830 | 16S RNA fragments |
| 99 | 4229828 | + | 17/17 | *pepE* | 4229453 | 4230142 | - |  | Antisense RNAs |
| 100 | **4236699** | - | 22/22 | *yjbF* | 4236262 | 4236900 | + |  | Antisense RNAs |
| 101 | 4318944 | + | 29/28 | *phnJ* | 4318761 | 4319606 | - |  | Antisense RNAs |
| 102 | **4347120** | - | 18/18 | *fumB* | 4345680 | 4347326 | - |  | mRNA fragments |
| 103 | 4420549 | - | 20/20 | *ulaA* | 4419980 | 4421377 | + |  | Antisense RNAs |
| 104 | 4502156 | - | 17/17 | *insG* | 4502103 | 4503431 | - | 4502190 | mRNA fragments |

The Table shows the positional coordinates in the *E. coli* genome for all the peaks found in the combined set of RNAs secreted by *E. coli* in experiments Eco_Rhod_1, Eco_Rhod_2, Eco_Rhod_3 (Table 1 in the main text), containing at least 16 sequence reads. The positions of the peaks overlapping with Supplementary Table 2 are shown in bold. “Str.” in the third column denotes the strand. The fourth column shows the total number of reads in the peak before and after removing of reads matching to the *R. rubrum* genome. Genome annotation was taken from RegulonDB (http://regulondb.ccg.unam.mx). The search for transcription start points (TSP) was done within 100 bp upstream from the 5’-end of the detected oligonucleotides using the PlatProm promoter finder (http://mathcell.ru/model6.php?l=en, Shavkunov et al. 2009). Their genomic coordinates are in bold if the corresponding promoters are indicated in RegulonDB. “PI” in this column means the presence of a *Promoter Island* in the region with multiple sigma-70 promoters on both strands, from which the detected oligonucleotides can be transcribed (described in Shavkunov et al. 2009). “MPI” means the presence of a Mixed Promoter Island in the region containing multiple promoters with different sigma-specificity (described in Panyukov et al. 2013).
