# Supplementary material for: Suppression of Escherichia coli Growth Dynamics via RNAs Secreted by Competing Bacteria
Source: Front Mol Biosci. 2021 Apr 15;8:609979. doi: 10.3389/fmolb.2021.609979 (PMC8082180; doi:10.3389/fmolb.2021.609979)
Supplement: Supplementary Table 4 — Genomic distribution of oligonucleotides dominant in the extracellular RNA fraction of mixed population E. coli MG1655–P. copri. [file Table_4.docx]

**Supplementary Table 4. Genomic location of oligonucleotides dominant in the extracellular RNA fraction of mixed population *E. coli* MG1655 – *P. copri***

|  | Position of the 5’-end | Str. | Number  of  reads in the peak | Associated genes | | | | TSP | Type of genomic  loci |
| --- | --- | --- | --- | --- | --- | --- | --- | --- | --- |
|  |  |  |  | Gene name | 5'-end  position | 3'-end  position | Str. |  |  |
| 1 | 88622 | + | 47/47 | *cra* | 88028 | 89032 | + |  | mRNA fragments |
| 2 | 207592 | + | 211/211 | *dnaE* | 205126 | 208608 | + | 207598 | mRNA fragments |
| 3 | **251076** | + | 34/34 | *dinB* | 250898 | 251953 | + |  | mRNA fragments |
| 4 | 331439 | - | 28/28 | *betT* | 329463 | 331496 | + |  | Antisense RNAs |
| 5 | 417760 | + | 39/39 | *phoB* | 417142 | 417831 | + |  | mRNA fragments |
| 6 | 441181 | + | 23/23 | *xseB* | 441101 | 441343 | - |  | Antisense RNAs |
| 7 | **499830** | + | 86/86 | *aes* | 499014 | 499973 | - | 499832 | Antisense RNAs |
| 8 | 545081 | - | 24/24 | *allC* | 544057 | 545292 | - | 545075 | mRNA fragments |
| 9 | **572550** | - | 61/48 | *ybcN* | 572466 | 572921 | + |  | Antisense RNAs |
| 10 | 622088 | - | 23/23 | *fepD* | 621185 | 622189 | - | 622123 | mRNA fragments |
| 11 | 631206 | - | 27/0 | *cstA* | 629894 | 631999 | + |  | Antisense RNAs |
| 12 | 653336 | + | 22/22 | *dpiB* | 652235 | 653893 | + |  | mRNA fragments |
| 13 | 751571 | + | 24/24 | *ybgQ* | 749722 | 752169 | - | 751564 | Antisense RNAs |
| 14 | 780597 | + | 43/0 | *lysT* | 780554 | 780629 | + | **780512** | tRNA 3'-end  (tRNA sample) |
|  | 780598 |  |  |  |  |  |  |  |  |
| 15 | 780886 | + | 43/0 | *lysW* | 780843 | 780918 | + |  | tRNA 3'-end |
|  | 780887 |  |  |  |  |  |  |  |  |
| 16 | 781190 | + | 43/0 | *lysY* | 781147 | 781222 | + |  | tRNA 3'-end |
|  | 781191 |  |  |  |  |  |  |  |  |
| 17 | 781412 | + | 43/0 | *lysZ* | 781369 | 781444 | + |  | tRNA 3'-end |
|  | 781413 |  |  |  |  |  |  |  |  |
| 18 | 781620 | + | 43/0 | *lysQ* | 781577 | 781652 | + |  | tRNA 3'-end |
|  | 781621 |  |  |  |  |  |  |  |  |
| 19 | **789928** | + | 22/0 | *galK* | 788831 | 789979 | - |  | Intergenic |
|  |  |  |  | *galT* | 789983 | 791029 | - |  |  |
| 20 | 816397 | - | 23/23 | *ybhK* | 815739 | 816647 | - |  | mRNA fragments |
| 21 | **836050** | - | 65/65 | *ybiB* | 835248 | 836210 | + |  | Antisense RNAs |
| 22 | 837078 | + | 30/0 | *hcxB* | 836351 | 837436 | + |  | mRNA fragments |
| 23 | **837243** | - | 47/47 | *hcxB* | 836351 | 837436 | + | 837268 | Antisense RNAs |
| 24 | **851867** | + | 55/55 | *opgE* | 851014 | 852597 | - |  | Antisense RNAs |
| 25 | **858567** | - | 49/0 | *ybiU* | 857796 | 859061 | - |  | mRNA fragments |
| 26 | **911195** | - | 45/45 | *hcr* | 911182 | 912150 | - |  | mRNA 3'-end |
| 27 | 940369 | + | 33/33 | *serS* | 939428 | 940720 | + |  | mRNA fragments |
| 28 | 958192 | + | 35/35 | *serC* | 957653 | 958741 | + |  | mRNA fragments |
| 29 | 990455 | - | 58/58 | *pncB* | 989154 | 990356 | - | 990476 | Intergenic |
|  |  |  |  | *pepN* | 990622 | 993234 | + |  |  |
| 30 | 1031651 | - | 66/66 | *serT* | 1031625 | 1031712 | - | **1031717** | tRNA fragment |
| 31 | 1078150 | + | 40/40 | *putA* | 1074920 | 1078882 | - |  | Antisense RNAs |
| 32 | 1143231 | - | 45/45 | *rne* | 1141182 | 1144367 | - |  | mRNA fragments |
| 33 | **1305928** | + | 38/13 | *oppF* | 1305764 | 1306768 | + |  | mRNA fragments |
| 34 | 1334865 | + | 25/25 | *cysB* | 1333855 | 1334829 | + | PI | Intergenic |
|  |  |  |  | *ymiA* | 1335148 | 1335288 | + |  |  |
| 35 | 1335335 | - | 142/142 | *yciX* | 1335291 | 1335458 | + |  | Antisense RNAs |
| 36 | 1347136 | + | 28/28 | *rnb* | 1346978 | 1348912 | - | 1347092 | Antisense RNAs |
| 37 | 1382181 | + | 51/51 | *ompG* | 1381947 | 1382852 | + | 1382150 | mRNA fragments |
| 38 | 1506475 | - | 44/31 | *sutR* | 1506172 | 1506708 | + |  | Antisense RNAs |
| 39 | 1539874 | - | 53/53 | *narZ* | 1538850 | 1542590 | + |  | Antisense RNAs |
| 40 | 1547114 | - | 32/0 | *yddG* | 1546288 | 1547169 | - | 1547159 | mRNA fragments |
| 41 | 1570289 | + | 44/44 | *gadC* | 1568954 | 1570489 | - | 1570282 | Antisense RNAs |
| 42 | **1694930** | - | 38/38 | *uidA* | 1694260 | 1696071 | - | 1694989 | mRNA fragments |
| 43 | 1703551 | - | 24/24 | *ydgJ* | 1703268 | 1704308 | - | 1703628 | mRNA fragments |
| 44 | **1711244** | - | 23/22 | *rsxE* | 1710828 | 1711523 | + |  | Antisense RNAs |
| 45 | 1761543 | + | 39/39 | *sufD* | 1760520 | 1761791 | - |  | Antisense RNAs |
| 46 | 1864586 | - | 40/40 | *yeaD* | 1863850 | 1864734 | + | 1864616 | Antisense RNAs |
| 47 | **1931024** | + | 37/29 | *purT* | 1930881 | 1932059 | + |  | mRNA fragments |
| 48 | 1962767 | - | 32/32 | *flhE* | 1962580 | 1962972 | - |  | mRNA fragments |
| 49 | 1990910 | - | 64/64 | *tyrP* | 1989681 | 1990892 | + | 1990970 | Intergenic |
|  |  |  |  | *yecA* | 1990954 | 1991619 | - |  |  |
| 50 | 2152536 | + | 39/39 | *yegL* | 2152469 | 2153128 | - |  | Antisense RNAs |
| 51 | **2160237** | + | 62/62 | *mdtC* | 2158386 | 2161463 | + | 2160171 | mRNA fragments |
| 52 | **2299478** | - | 34/34 | *napH* | 2298715 | 2299578 | - |  | mRNA fragments |
| 53 | **2309398** | - | 54/54 | *ada* | 2309341 | 2310405 | - | 2309454 | mRNA fragments |
| 54 | 2382830 | + | 38/1 | *elaD* | 2382713 | 2383924 | + | 2382810 | mRNA fragments |
| 55 | 2385392 | - | 81/81 | *yfbK* | 2383995 | 2385722 | - |  | mRNA fragments |
| 56 | 2521296 | + | 43/0 | *lysV* | 2521253 | 2521328 | + |  | tRNA 3'-end  (tRNA sample) |
|  | 2521297 |  |  |  |  |  |  |  |  |
| 57 | **2592891** | - | 55/47 | *ypfN* | 2592762 | 2592962 | + |  | Antisense RNAs |
| 58 | 2609237 | + | 29/29 | *hyfG* | 2608487 | 2610154 | + | 2609142 | mRNA fragments |
| 59 | **2631172** | - | 126/125 | *guaA* | 2630958 | 2632535 | - |  | mRNA fragments |
| 60 | **2674086** | + | 43/42 | *yphB* | 2673816 | 2674688 | - |  | Antisense RNAs |
|  | **2674087** | + | 69/1 |  |  |  |  |  |  |
|  | 2674088 | + | 27/0 |  |  |  |  |  |  |
| 61 | **2675086** | - | 62/0 | *yphC* | 2674700 | 2675761 | - |  | mRNA fragments |
| 62 | 2700429 | - | 57/0 | *shoB* | 2700117 | 2700197 | - | 2700429 | Intergenic |
|  |  |  |  | *ohsC* | 2700520 | 2700596 | + |  |  |
| 63 | 2718787 | + | 38/38 | *trxC* | 2718735 | 2719154 | + | 2718737 | mRNA fragments |
| 64 | 2723770 | + | 49/0 | *pssA* | 2722727 | 2724082 | + | 2723717 | mRNA fragments |
| 65 | 2734183 | - | 28/13 | *ryfD* | 2734153 | 2734295 | - | 2734201 | sRNA fragment |
| 66 | **2781826** | + | 41/41 | *ypjA* | 2778146 | 2782726 | - | 2781794 | Antisense RNAs |
| 67 | **2822502** | + | 25/25 | *pncC* | 2823849 | 2824346 | - |  | Antisense RNAs |
| 68 | 2945808 | - | 24/24 | *tcdA* | 2945036 | 2945842 | - | 2945899 | mRNA fragments |
| 69 | 3005539 | + | 47/47 | *ygeV* | 3004008 | 3005786 | - | 3005529 | Antisense RNAs |
| 70 | **3047984** | - | 93/93 | *gcvP* | 3046168 | 3049041 | - | 3048006 | mRNA fragments |
| 71 | 3068644 | - | 25/25 | *argO* | 3068173 | 3068808 | - | 3068691 | mRNA fragments |
| 72 | 3110417 | + | 39/39 | *pheV* | 3110366 | 3110441 | + | **3110363** | tRNA 3'-end |
| 73 | **3111478** | - | 42/0 | *yghE* | 3111128 | 3111988 | - |  | mRNA fragments |
| 74 | 3210793 | + | 36/31 | *rpsU* | 3210781 | 3210996 | + | **3210735** | mRNA fragments |
| 75 | **3289146** | + | 31/1 | *yraJ* | 3288814 | 3291330 | + | 3289137 | mRNA fragments |
| 76 | 3309002 | - | 24/24 | *nlpI* | 3308040 | 3308924 | - |  | Intergenic |
|  |  |  |  | *pnp* | 3309033 | 3311168 | - |  |  |
| 77 | **3344580** | + | 27/27 | *lptB* | 3343944 | 3344669 | + |  | mRNA fragments |
| 78 | 3419010 | - | 26/26 | *yhdV* | 3418390 | 3418611 | + | 3419014 | Intergenic |
|  |  |  |  | *yhdW* | 3419042 | 3420066 | + |  |  |
| 79 | **3450951** | + | 265/264 | *rplB* | 3450543 | 3451364 | - | 3450950 | Antisense RNAs |
| 80 | **3467240** | + | 67/67 | *chiA* | 3467160 | 3469853 | - | 3467199 | Antisense RNAs |
| 81 | **3513124** | - | 41/0 | *trpS* | 3512634 | 3513638 | - |  | mRNA fragments |
| 82 | **3533981** | -/+ | 50/44 | *pck* | 3532818 | 3534440 | + |  | Antisense RNAs |
| 83 | **3543448** | - | 35/35 | *rpnA* | 3543167 | 3544045 | + |  | Antisense RNAs |
| 84 | 3562866 | + | 30/0 | *glpD* | 3562013 | 3563518 | + |  | mRNA fragments |
| 85 | 3706872 | - | 30/28 | *dppA* | 3706098 | 3707705 | - |  | mRNA fragments |
| 86 | 3724344 | + | 23/23 | *glyS* | 3722328 | 3724397 | - |  | Antisense RNAs |
| 87 | 3739115 | + | 28/28 | *malS* | 3737497 | 3739527 | + |  | mRNA fragments |
| 88 | 3800488 | -/+ | 41/41 | *waaY* | 3800267 | 3800965 | - | MPI | mRNA fragments |
| 89 | **3836960** | + | 57/39 | *setC* | 3836953 | 3838137 | + | PI | mRNA 5'-end |
| 90 | 3853756 | - | 61/3 | *tisB* | 3853553 | 3853642 | + | 3853770 | Intergenic |
|  |  |  |  | *ysdE* | 3853766 | 3853840 | - |  |  |
| 91 | 3863351 | - | 75/75 | *glvC* | 3862497 | 3863603 | - | 3863433 | mRNA fragments |
| 92 | 3878689 | + | 39/1 | *gyrB* | 3877705 | 3880119 | - |  | Antisense RNAs |
| 93 | **3918588** | - | 23/23 | *atpA* | 3918316 | 3919857 | - |  | mRNA fragments |
| 94 | **3922927** | - | 63/52 | *atpI* | 3922060 | 3922440 | - | PI | Intergenic |
|  |  |  |  | *rsmG* | 3923057 | 3923680 | - |  |  |
| 95 | 4104684 | - | 83/83 | *cpxA* | 4103602 | 4104975 | - |  | mRNA fragments |
| 96 | 4107960 | + | 31/31 | *pfkA* | 4107552 | 4108514 | + |  | mRNA fragments |
| 97 | 4116803 | + | 50/50 | *glpK* | 4115714 | 4117222 | - |  | Antisense RNAs |
| 98 | 4142794 | - | 61/61 | *frwC* | 4142530 | 4143609 | + |  | Antisense RNAs |
| 99 | 4215853 | - | 42/31 | *aceB* | 4215478 | 4217079 | + | 4215900 | Antisense RNAs |
| 100 | 4218882 | - | 23/23 | *aceK* | 4218596 | 4220332 | + |  | Antisense RNAs |
| 101 | 4246557 | + | 34/34 | *malE* | 4245229 | 4246419 | - |  | Intergenic |
|  |  |  |  | *malK* | 4246784 | 4247899 | + |  |  |
| 102 | 4282817 | + | 29/29 | *yjcF* | 4281783 | 4283075 | - | PI | Antisense RNAs |
| 103 | **4347120** | - | 25/0 | *fumB* | 4345680 | 4347326 | - |  | mRNA fragments |
| 104 | 4362575 | - | 39/39 | *pheU* | 4362551 | 4362626 | - | **4362629** | mRNA fragments |
| 105 | 4384547 | + | 26/23 | *yjeM* | 4383839 | 4385341 | + | MPI | mRNA fragments |
| 106 | 4463720 | - | 45/45 | *treC* | 4463054 | 4464709 | - |  | mRNA fragments |
| 107 | 4502156 | - | 29/29 | *insG* | 4502103 | 4503431 | - | 4502190 | mRNA fragments |
| 108 | 4521069 | - | 40/21 | *yjhF* | 4520671 | 4522020 | - |  | mRNA fragments |
| 109 | 4618019 | - | 53/53 | *deoC* | 4617323 | 4618102 | + |  | Antisense RNAs |

The Table shows the positional coordinates in the *E. coli* genome for all the peaks found in the combined set of RNAs secreted by *E. coli* in experiments Eco_Prevot_1, Eco_Prevot_2, Eco_Prevot_3 and Eco_Prevot_4 (Table 1 in the main text), containing more than 20 sequence reads in the peak maxima. The positions of the peaks overlapping with Supplementary Table 2 are shown in bold. “-/+” in the third column (Str. = strand) indicates that along with main oligonucleotides derived from the “-” strand, there are complementary transcripts from the “+” strand. The fourth column shows the total number of reads in the peak before and after removing of reads matching to the *R. rubrum* genome. Genome annotation was taken from RegulonDB (http://regulondb.ccg.unam.mx). The search for transcription start points (TSP) was done within 100 bp upstream from the 5’-end of the detected oligonucleotides using the PlatProm promoter finder (http://mathcell.ru/model6.php?l=en, Shavkunov et al. 2009). Their genomic coordinates are in bold if the corresponding promoters are indicated in RegulonDB. “PI” in this column means the presence of a *Promoter Island* in the region with multiple sigma-70 promoters on both strands, from which the detected oligonucleotides can be transcribed (described in Shavkunov et al. 2009). “MPI” means the presence of a Mixed Promoter Island in the region containing multiple promoters with different sigma-specificity (described in Panyukov et al. 2013).
